# Development of Biofortified Maize Hybrids through Marker-Assisted Stacking of β*-Carotene Hydroxylase, Lycopene-*ε*-Cyclase* and *Opaque2* Genes

**DOI:** 10.3389/fpls.2018.00178

**Published:** 2018-02-20

**Authors:** Rajkumar U. Zunjare, Firoz Hossain, Vignesh Muthusamy, Aanchal Baveja, Hema S. Chauhan, Jayant S. Bhat, Nepolean Thirunavukkarasu, Supradip Saha, Hari S. Gupta

**Affiliations:** ^1^Division of Genetics, ICAR-Indian Agricultural Research Institute, New Delhi, India; ^2^Regional Research Centre, ICAR-Indian Agricultural Research Institute, Dharwad, India; ^3^Division of Agricultural Chemicals, ICAR-Indian Agricultural Research Institute, New Delhi, India

**Keywords:** provitamin A, *crtRB1*, *lcyE*, QPM, marker-assisted selection, biofortification

## Abstract

Traditional yellow maize though contains high kernel carotenoids, the concentration of provitamin A (proA) is quite low (<2 μg/g), compared to recommended level (15 μg/g). It also possesses poor endosperm protein quality due to low concentration of lysine and tryptophan. Natural variant of *crtRB1* (β*-carotene hydroxylase*) and *lcyE* (*lycopene-*ε*-cyclase*) cause significant enhancement of proA concentration, while recessive allele, *opaque2* (*o2*) enhances the level of these amino acids. Development of biofortified maize enriched in proA, lysine and tryptophan thus holds significance in alleviation of micronutrient malnutrition. In the present study, marker-assisted stacking of *crtRB1, lcyE* and *o2* was undertaken in the genetic background of four maize hybrids (HQPM1, HQPM4, HQPM5, and HQPM7) popularly grown in India. HP704-22 and HP704-23 were used as donors, while four elite QPM parents viz., HKI161, HKI163, HKI193-1, and HKI193-2 were used as recipients. *CrtRB1* showed severe segregation distortion, while *lcyE* segregated as per the expectation. Recovery of recurrent parent genome (RPG) among selected backcross progenies ranged from 89 to 93%. Introgressed progenies possessed high concentration of proA (7.38–13.59 μg/g), compared to 1.65–2.04 μg/g in the recurrent parents. The reconstituted hybrids showed an average of 4.5-fold increase in proA with a range of 9.25–12.88 μg/g, compared to original hybrids (2.14–2.48 μg/g). Similar plant-, ear-, and grain- characteristics of improved versions of both inbreds and hybrids were observed when evaluated with their respective original versions. Mean lysine (0.334%) and tryptophan (0.080%) of the improved hybrids were *at par* with the original versions (lysine: 0.340%, tryptophan: 0.083%). Improved hybrids also possessed similar grain yield potential (6,301–8,545 kg/ha) with their original versions (6,135–8,479 kg/ha) evaluated at two locations. This is the first study of staking *crtRB1*-, *lcyE*-, and *o2*-, favorable alleles in single genetic background. The improved inbreds can be effectively used as potential donor for independent and/or simultaneous introgression of *crtRB1, lcyE*, and *o2* in the future breeding programme. These biofortified maize hybrids, rich in proA, lysine and tryptophan will hold great promise for nutritional security.

## Introduction

Micronutrient malnutrition popularly known as “hidden hunger” is a serious health problem worldwide, particularly in the under-developed and developing countries (Bouis and Saltzman, 2017). Nearly two billion people suffer from deficiency of micronutrients, while 815 million people are under-nourished (Global Nutrition Report, 2017). Among micronutrients, vitamin A plays, key role in human metabolism. This deficiency lead to visual blindness which may cause eye sight damage to millions preschool-age children. According to HarvestPlus, nearly 20 million pregnant women are vitamin A deficient, while out of which about one-third are clinically night-blind. There are about one-half of these cases occur in India with severe form of vision impairment. The deficiency of lysine and tryptophan leads to fatigue, delayed growth, loss of appetite, depression, anxiety in children (Nuss and Tanumihardjo, 2010; Jompuk et al., 2011). Moreover, unbalanced protein in the diet leads to protein energy malnutrition (PEM) that affects more than a billion people across the world (Bain et al., 2013). The adoption of quality protein maize (QPM) varieties possessing balanced protein due to higher lysine and tryptophan which has shown significant promise in solving problem of PEM across the world (Nyakurwa et al., 2017).

Cereals are rich source of energy, but lacking the required content of micronutrients (Nuss and Tanumihardjo, 2010). Genetic enhancement of micronutrient in crops through plant breeding known as “biofortification” which is a cost-effective and sustainable process, where micronutrients reach the target group in their natural form (Pfeiffer and McClafferty, 2007; Gupta et al., 2015; Neeraja et al., 2017). Maize occupies an important position in the world economy. It along with rice and wheat provides at least 30% of the food calories to more than 4.5 billion people in 94 developing countries, besides serving as a major component of animal feed (Shiferaw et al., 2011). In India, maize is the third most cereal after rice and wheat, and used as an important source of both food and feed (Yadav et al., 2015). Normal maize protein contains lower level of lysine (0.16–0.26%) and tryptophan (0.02–0.06%) which is less than half of the recommended dose specified for human nutrition (Bjarnason and Vasal, 1992; Vivek et al., 2008). Further, traditional yellow maize contains enough kernel carotenoids as compared to other cereals. However, it is predominated by non-proA fractions and contains only 0.25–2.50 μg/g of proA carotenoids which is far below the nutritional requirement (15 μg/g) for humans (Pixley et al., 2013).

Favorable alleles of *lycopene* ε*-cyclase* (*lcyE*) and β*-carotene hydroxylase1* (*crtRB1*) genes causes enhancement in proA in maize (Harjes et al., 2008; Yan et al., 2010; Babu et al., 2013). The recessive *opaque2* (*o2*) allele enhances endosperm lysine and tryptophan by almost 2-folds (Mertz et al., 1964). Marker-assisted selection (MAS) using very low expensive DNA markers helps in stacking of multiple target genes into a genetic background without progeny testing (Das et al., 2017). It also significantly reduces the breeding cycles required to reconstitute the recurrent parent genome (RPG) (Gupta et al., 2013). Further, high cost of HPLC (High Performance Liquid Chromatography) analyses for estimation of micronutrients among individuals of segregating populations could be avoided through usage of molecular markers. The successful examples of application MAS in development of nutritious maize hybrids in India have been the commercial release of “Vivek QPM9” (Gupta et al., 2013), “Pusa Vivek QPM9 Improved” (Muthusamy et al., 2014), “Pusa HM4 Improved,” “Pusa HM8 Improved,” and “Pusa HM9 Improved” (Hossain et al., 2017). Lysine and tryptophan rich QPM hybrids of late maturity so far released in the India do not contain recommended level of proA concentration. The present study was thus aimed to (i) stack favorable alleles of *crtRB1, lcyE* and *opaque2* genes into elite inbreds/hybrids by using marker-assisted backcross breeding (MABB) and (ii) evaluate the MABB-derived –inbreds/hybrids for nutritional quality, agronomic and yield related traits.

## Materials and methods

### Plant materials

The parental inbreds viz., HKI161, HKI163, HKI193-1, and HKI193-2 of four QPM hybrids, [HQPM1 (HKI193-1 × HKI163), HQPM4 (HKI193-2 × HKI161), HQPM5 (HKI163 × HKI161) and HQPM7 (HKI193-1 × HKI161)], were targeted for enrichment of micronutrients. The popular and commercial maize hybrids are adapted to diverse agro-ecologies of India (Table 1). Recurrent parents were crossed with donor lines and four crosses viz., cross-I (HKI161 × HP704-23), cross-II (HKI163 × HP704-22), cross-III (HKI193-1 × HP704-23), cross-IV (HKI193-2 × HP704-22) were attempted to stack *crtRB1, lcyE*, and *o2* in the genetic background of recurrent parents. The pedigree information of the recurrent parents and donors is given in Table S1.

**Table 1:** Details of popular commercial QPM hybrids targeted for provitamin A enrichment.

**S. No**.	**Hybrid**	**Parental lines**	**Maturity group**	**Maturity (days)**	**Year of release**	**Area of adaptation**
1.	HQPM1	HKI193-1 × HKI163	Late	88-90	2005	Zone-II, III, IV, V
2.	HQPM4	HKI193-2 × HKI161	Late	95-97	2010	Zone-II, III, IV, V
3.	HQPM5	HKI163 × HKI161	Late	92-93	2007	Zone-II, III, IV, V
4.	HQPM7	HKI193-1 × HKI161	Late	96-97	2008	Zone-IV

*Zone II, North Western Plain Zone; Zone III, North Eastern Plain Zone; Zone IV, Peninsular Zone; Zone V, Central Western Zone*.

### Generation of backcross-and self-progenies

**Figure 1:**
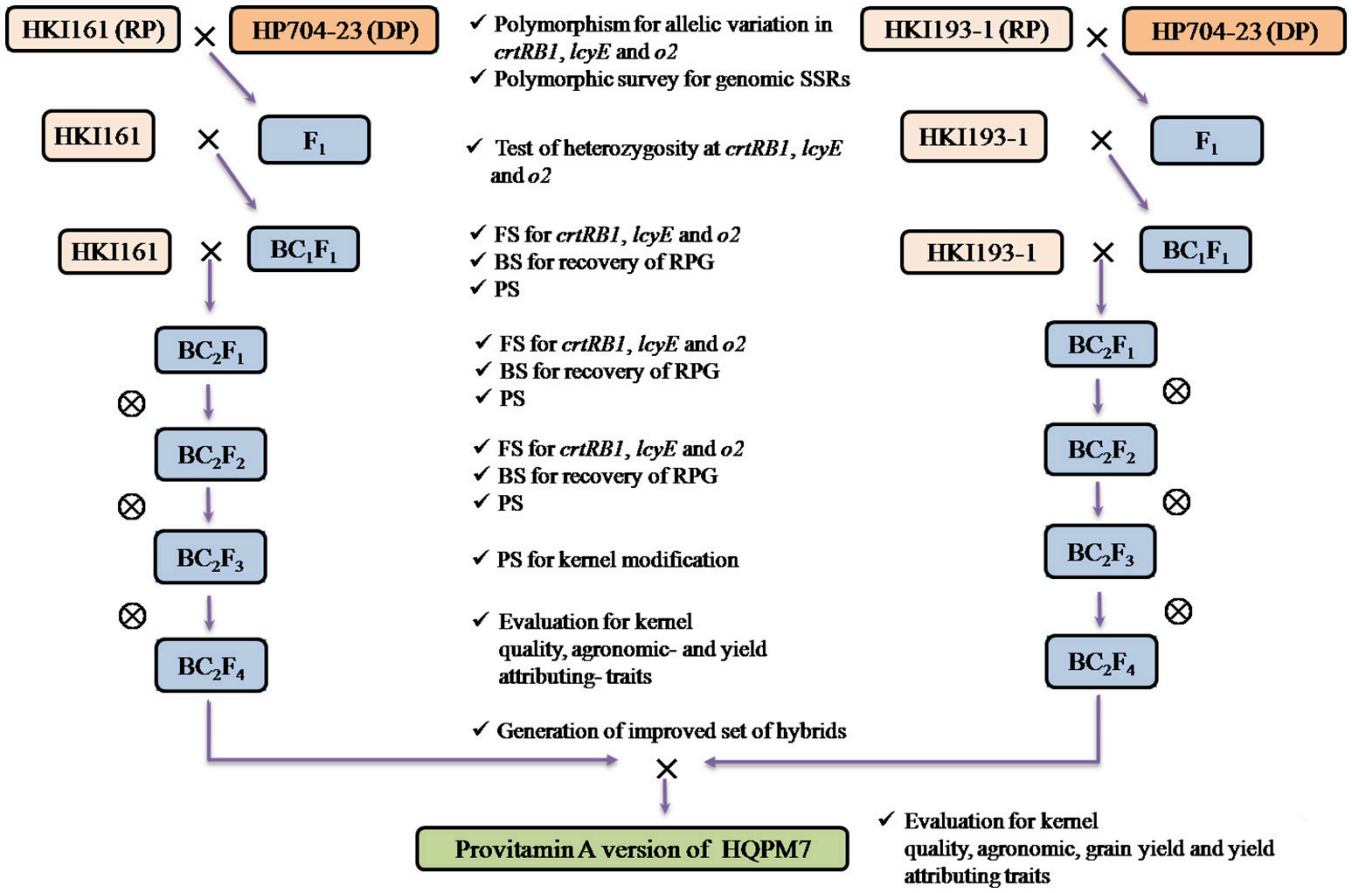
Marker-assisted backcross breeding scheme followed for development of provitamin A, lysine and tryptophan rich maize hybrid (e.g., HQPM7). RP, Recurrent Parent; DP, Donor Parent.

Backcross- and self- generations which were grown at different locations are described in Table S2, and the MABB scheme followed is represented as Figure 1. The recipients and donors showing polymorphism for gene-based markers were crossed during *rainy* season (July-November 2012) at IARI, New Delhi (28°089N, 77°129E, 229 MSL). Hybridity of the F_1_s was tested using gene-based markers, and the true F_1_s were backcrossed to their corresponding recurrent parent during *winter* season (December, 2012-April, 2013) at Winter Nursery Centre (WNC), Hyderabad (17°199N, 78°249E, 542.6 MSL). The BC_1_F_1_ progenies were grown at IARI, New Delhi during *rainy* season (2013), and foreground selection was carried out (Figure S1). The foreground positive plants with high recovery of RPG (RPG) and maximum phenotypic similarity were further backcrossed to the recurrent parent. The BC_2_F_1_ progenies were grown at WNC, Hyderabad during *winter* season (2013-14), and were subjected to foreground-, background- and phenotypic selection. The BC_2_F_2_ progenies were raised during *rainy* season (2014) at IARI, New Delhi. Foreground positive plants homozygous for all genes were subjected to background- and phenotypic- selection. The selected plants were subsequently self-pollinated to generate BC_2_F_3_ and BC_2_F_4_ progenies (Table S2).

### Marker-assisted foreground selection

Three SSR markers based on *o2* gene, viz., *phi057, phi112*, and *umc1066* were screened to distinguish the parental lines, of which, *phi057* marker revealed polymorphic pattern between recipients and donors (Gupta et al., 2013). Polymerase chain reaction (PCR) amplification for SSRs was followed as per Hossain et al. (2017). Four percent of Seakem LE agarose (Lonza, Rockland, ME USA) gel was used for electrophoretic separation of PCR products at 120 V for 3–5 h along with 100 bp DNA ladder (MBA-Fermentas). Gene-based *InDel* marker present in 3'TE and 5'TE region of *crtRB1* and *lcyE* were used for foreground selection (Harjes et al., 2008; Yan et al., 2010; Figure S1; Table 2). PCR mediated amplification of *crtRB1* and *lcyE* was performed using protocol standardized at Maize Genetics Unit, IARI (Zunjare et al., 2017a). Agarose of 1.5% concentration (Lonza, Rockland, ME USA) was used for separating the amplicon at 120 V for 2–3 h along with 100 bp DNA ladder (MBA-Fermentas). The amplified products were visualized using a gel documentation system (Alpha Innotech, California, USA) and scored for the presence and absence of designated allele.

**Table 2:** Details of gene-based markers used for foreground selection in MABB.

**S. No**.	**Gene**	**Bin location**	**Marker**	**Primer Sequence (5′–3′)**	**Primer**	**References**
1.	*crtRB1*	10.05	3′TE InDel	ACACCACATGGACAAGTTCG	Forward	Yan et al., 2010
				ACACTCTGGCCCATGAACAC	Reverse1	
				ACAGCAATACAGGGGACCAG	Reverse2	
2.	*lcyE*	8.05	5′TE InDel	AAGCAGGGAAGACATTCCAG	Forward	Babu et al., 2013
				GAGAGGGAGACGACGAGACAC	Reverse	
3.	*opaque2*	7.01	*phi057*	CTCATCAGTGCCGTCGTCCAT	Forward	Gupta et al., 2013
				CAGTCGCAAGAAACCGTTGCC	Reverse	

### Marker-assisted background selection

SSRs with near uniform coverage across 10 chromosomes of maize genome were used for polymorphism survey between the respective recurrent and donor genotypes (Table 3, Table S3). The primer sequences of the SSRs were retrieved from the maize genome database (www.maizegdb.org) and were custom synthesized (Sigma Tech., USA). PCR amplification and scoring of amlicons was undertaken as per Hossain et al. (2017). The markers which were polymorphic between the recurrent and their respective donor parents were employed for recovering the RPG in individuals of BC_1_F_1_, BC_2_F_1_, and BC_2_F_2_ generations.

**Table 3:** Number of screened SSRs and percentage polymorphism observed in four crosses.

**LG**	**No. of markers screened**	**HKI161** × **HP704-23**		**HKI163** × **HP704-22**		**HKI193-1** × **HP704-23**		**HKI193-2** × **HP704-22**	
		**NP**	**Pol. (%)**	**NP**	**Pol. (%)**	**NP**	**Pol. (%)**	**NP**	**Pol. (%)**
1	17	11	64.71	13	76.47	11	64.71	12	70.59
2	21	10	47.62	12	57.14	12	57.14	12	57.14
3	29	17	58.62	12	41.38	18	62.07	16	55.17
4	23	11	47.83	14	60.87	14	60.87	16	69.57
5	21	13	61.90	14	66.67	15	71.43	13	61.90
6	23	13	56.52	13	56.52	16	69.57	13	56.52
7	19	9	47.37	17	89.47	12	63.16	13	68.42
8	15	7	46.67	11	73.33	9	60.00	6	40.00
9	19	12	63.16	10	52.63	14	73.68	11	57.89
10	21	11	52.38	11	52.38	12	57.14	12	57.14
Total	208	114	54.81	127	61.06	133	63.94	124	59.62

*LG, Linkage group; NP, No. of observed polymorphic markers; Pol. (%) ,Polymorphism percentage*.

### Phenotypic selection

Selection of plant-, ear-, and grain-characteristics was performed among the individuals of each backcross- and self- generations for their similarity with their respective recurrent parents. The harvested BC_2_F_3_ seeds from the introgressed progenies were subjected to standard light box test along with the original recurrent parental seeds to measure the intensity of opaqueness (Hossain et al., 2008). The seeds with similar degree of opaqueness of the original inbreds were forwarded for further generation and the reconstitution of hybrids (Vivek et al., 2008; Gupta et al., 2013).

### Analysis of provitamin a, lysine, and tryptophan

The selfed seeds of BC_2_F_4_ plants (two BC_2_F_3_ populations for HKI163) were utilized for biochemical analysis. The selfed ears were harvested at moisture level 12–14%, and then cleaned and dried under the shade. The equal amount of grains shelled from ears of same families was bulked together, and the samples thus drawn were stored in ambient temperature (22–26°C) for 2 months before biochemical analysis.

The extraction of β-carotene (BC) and β-cryptoxanthin (BCX) from maize seeds was carried out using procedures described by Kurilich and Juvik (1999) and Vignesh et al. (2012). Quantification of the BC and BCX was carried out with a Dionex Ultimate 3000 UHPLC System (Ultra High Performance Liquid Chromatography; Thermo Scientific, Massachusetts, USA). Samples were eluted through Carotenoid C_30_ column (5 μm, 4.6 × 250 mm, YMC) and detected with a diode array detector-3000 (RS). The mobile phase consisted of methanol: tert-butyl methyl ether (80:20, v/v), and the flow rate was 1 ml min^−1^. The standards of BC and BCX (Sigma Aldrich, USA) were used to make the regression curves and estimate the components with an absorbance at 450 nm. The proA concentration (μg/g on dry weight basis) was estimated as sum of BC plus half the BCX concentration (Babu et al., 2013).

The protocol standardized by Sarika et al. (2017) was followed to estimate lysine and tryptophan content of maize endosperm. Amino acids were estimated by using Dionex Ultimate 3000 UHPLC system (Thermo Scientific, Massachusetts, USA). The samples were eluted through Acclaim^TM^ 120 C_18_ column (5 μm, 120A°, 4.6 × 150 mm, Thermo Scientific) and detected with a RS photodiode array detector (PDA) with absorbance in 265 and 280 nm wavelength, respectively for lysine and tryptophan. Final concentration of the amino acids in each sample was estimated by standard regression using external standards (AAS 18-5 ML, Sigma Aldrich).

### Evaluation of introgressed inbreds

Twelve improved progenies (BC_2_F_3_/BC_2_F_4_) along with the respective recurrent parents were evaluated during *rainy* season (2015) at IARI Experimental Farm, New Delhi. Two-three plants per entry were self-pollinated, and selfed grains were analyzed for proA, lysine and tryptophan. Characters viz., days to 50% male flowering (MF), days to 50% female flowering (FF), plant height (PH), ear height (EH), ear length (EL), ear width (EW), number of rows (NR), number of kernels per row (NKR), and 100-seed weight (TW) were recorded from open pollinated plants.

### Evaluation of reconstitution of hybrids

Selected 12 (BC_2_F_4_/BC_2_F_5_) progenies of the four improved inbreds were used to reconstitute twelve F_1_ hybrids during *winter* season (2015-16) at WNC, IIMR, Hyderabad. Three versions of the reconstituted hybrids (-A, -B, and -C) and their corresponding original hybrids were evaluated in Randomized Complete Block Design (RCBD) with two replications at two diverse maize growing zones of the country viz., IARI Experimental Farm, New Delhi in Northern India and IARI Regional Research Centre, Dharwad (15°219N, 75°059E, 750 MSL) Karnataka in Southern India during *rainy* season of 2016. Two to three plants in each of the hybrid entries were self-pollinated. Since, proA (Vignesh et al., 2012), lysine and tryptophan (Pixley and Bjarnason, 2002) do not vary much across locations, selfed seeds from IARI Experimental Farm, New Delhi were used for analysis of quality traits. However, morphological traits viz., MF, FF, PH, EH, EL, EW, NR, NKR, TW, and grain yield (GY) were recorded in open pollinated plants at both the locations.

### Statistical analysis

The observed segregation pattern of *crtRB1* and *lcyE* across segregating populations (BC_1_F_1_, BC_2_F_1_, and BC_2_F_2_), and *o2* in BC_1_F_1_ generation of four crosses was tested for goodness of fit by χ^2^ analysis. The amplicons of markers used in background selection were scored as “A” for the recipient allele, “B” for the donor allele, and “H” for the heterozygous genotype. Recovery percentage of RPG was estimated using formula, RPG (%) = [A + (0.5H)/(A + B + H)] × 100 (Benchimol et al., 2005). The recovery of RPG among selected backcross-derived progenies was also established using Graphical Geno Types (GGT) version 3.0 (Van-Berloo, 1999). Graphical representation based on mean of improved proA, lysine and tryptophan was ascertained by Microsoft Excel (2013). Agronomic and biochemical data of hybrids were analyzed using Windostat 8.5 software package (Khetan, 2005).

## Results

### Marker- and trait-polymorphism among parents

All the four recurrent parents (HKI161, HKI163, HKI193-1, and HKI193-2) revealed unfavorable allele (*C*^+^: 296 bp), while the donors possessed favorable allele (*C*: 543 bp) of *crtRB1* gene. Polymorphism test for *lcyE* revealed the presence of favorable allele (*L*: 650 bp) in two recurrent (HKI161 and HKI163) and two donor (HP704-22 and HP704-23) parents, while HKI193-1 and HKI193-2 possessed unfavorable allele (*L*^+^: 300 bp). All the recurrent parents possessed low proA concentration (HKI161: 2.04 μg/g, HKI163: 1.65 μg/g, HKI193-1: 1.84 μg/g, and HKI193-2: 1.74 μg/g), while donor parents possessed high proA concentration (HP704-22: 16.05 μg/g and HP704-23: 15.28 μg/g).Based on *phi057*, recessive allele of *o2* (165 bp) was present in all QPM recurrent parents while, the donors possessed dominant allele, *O2* (159 bp). The lysine (HKI161: 0.308%, HKI163: 0.347%, HKI193-1: 0.323%, and HKI193-2: 304%) and tryptophan (HKI161: 0.076%, HKI163: 0.082%, HKI193-1: 0.078%, and HKI193-2: 0.071%) content of recurrent parents was higher than their donor parents (Lysine, HP704-22: 0.176% and HP704-23: 0.192%; Tryptophan, HP704-22: 0.028% and HP704-23: 0.035%). A total of 114, 127, 133, and 124 polymorphic SSRs with polymorphism of 54.81, 61.06, 63.94, and 59.62% were observed in HKI161 × HP704-23, HKI163 × HP704-22, HKI193-1 × HP704-23, and HKI193-2 × HP704-22, respectively (Table 3, Table S3). The number of polymorphic markers in each chromosome ranged 7–17. These polymorphic markers were used for background selection for recovering the RPG in the backcross-derived populations.

### Marker-assisted stacking of *crtRB1 lcyE* and *o2*

**Figure 2:**
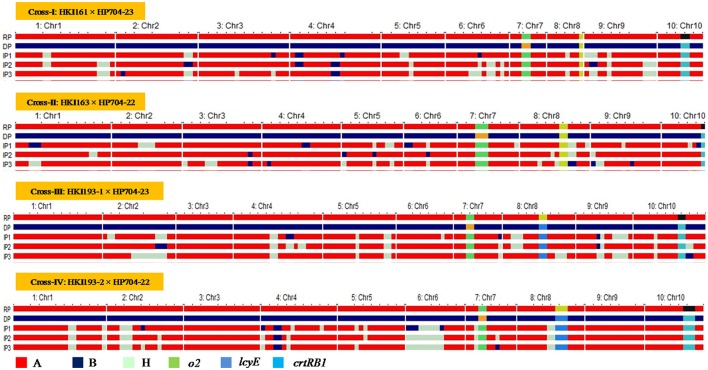
Graphical genotype of introgressed progenies across the four crosses. RP, Recurrent Parent; DP, Donor Parent; IP, Introgressed Progeny; Chr, Chromosome.

The hybridity test in F_1_ generation has confirmed the success of crossing of parental lines. In BC_1_F_1_ and BC_2_F_1_ populations, the range of 100–120 and 106–122 plants, respectively were subjected to foreground selection of *crtRB1* gene (Figure S1). The heterozygous progenies for *crtRB1* were then subjected to foreground selection of *lcyE*. The polymorphic pattern for *lcyE* was observed only for viz., HKI193-1 × HP704-23 and HKI193-2 × HP704-22. The progenies (*C*^+^*C/LL* in cross-I and -II, and *C*^+^*C/L*^+^*L* in cross-III and -IV) were further subjected to foreground selection for *o2* allele using *phi057*. The progenies with *o2* allele in homozygous state were selected in BC_1_F_1_. The population size used for analysis, segregation pattern, chi-square test results are mentioned in Table 4. Subsequently, foreground positive plants were analyzed for background selection using polymorphic markers. The recovery of RPG varied from 70.47 to 80.83% across four BC_1_F_1_, while RPG varied from 83.07 to 90.60% in the four BC_2_F_1_ were observed (Table 5). Stringent phenotypic selection was also applied considering plant- architecture, ear- and grain- related traits. Foreground selection was executed among the plants in cross-I, -III and –IV, respectively to identify plants of genotype *CC/LL/o2o2* (Table 3). For cross-II, two BC_2_F_2_ populations, BC_2_F_2_-I and BC_2_F_2_-II were raised in *rainy* season 2014 and *winter* season 2014-15, respectively. The RPG recovery in the selected plants ranged from 83.86 to 92.98% across four crosses (Table 4, Figure 2). Selection of morphological traits helped in deriving phenotypically similar progenies with their original versions. However, in case of HKI193-2-based progenies, *CC/LL/o2o2* possessed undesirable characteristics of tip opening of ear and irregular grain arrangements, thus were not selected. Instead, progenies of genetic constitution “*CC/L*^+^*L/o2o2*” with desirable characteristics was selfed to develop BC_2_F_3_ population in *winter* season 2014-15 to recover “*CC/LL/o2o2*” genotypes (Table S2).The segregation pattern of *crtRB1* locus showed deviation from the expected Mendelian ratio in all populations across the generations of four crosses, but *lcyE* segregated as per the expectation. *o2* gene was also showed Mendelian monohybrid pattern of inheritance except in cross-II.

**Table 4:** Segregation pattern of *crtRB1, lcyE* and *opaque2* in different backcross- and self- generations of the four crosses.

**Cross**	**Stage**	**N**	**C^+^C^+^**	**C^+^C**	**CC**	**df**	**χ^2^**	***P*-value**	**N**	**L^+^L^+^**	**L^+^L**	**LL**	**df**	**χ^2^**	***P*-value**	**N**	**O2o2**	**o2o2**	**df**	**χ^2^**	***P*-value**
HKI161 × HP704-23	BC_1_F_1_	120	73	47	–	1	5.63	0.0176^**^	47	–	–	47	–	–	–	47	26	21	1	0.53	0.46^ns^
	BC_2_F_1_	108	88	20	–	1	42.81	0.0001^**^	20	–	–	20	–	–	–	20	–	20	–	–	–
	BC_2_F_2_	104	46	37	21	2	20.67	0.0001^**^	21	–	–	21	–	–	–	21	–	21	–	–	–
HKI163 × HP704-22	BC_1_F_1_	120	84	36	–	1	19.2	0.0001^**^	36	–	–	36	–	–	–	36	24	12	1	4.00	0.04^*^
	BC_2_F_1_	106	82	24	–	1	31.73	0.0001^**^	24	–	–	24	–	–	–	24	–	24	–	–	–
	BC_2_F_2_-I	108	59	26	23	2	53.03	0.0001^**^	23	–	–	23	–	–	–	23	–	23	–	–	–
	BC_2_F_2_-II	101	48	36	17	2	28.90	0.0001^**^	17	–	–	17	–	–	–	17	–	17			
HKI193-1 × HP704-23	BC_1_F_1_	100	71	29	–	1	17.64	0.0001^**^	29	12	17	–	1	0.86	0.35^ns^	17	5	12	1	2.88	0.08^ns^
	BC_2_F_1_	116	89	27	–	1	33.13	0.0001^**^	27	15	12	–	1	0.33	0.56^ns^	12	–	12	–	–	–
	BC_2_F_2_	110	50	33	27	2	27.21	0.0001^**^	110	35	48	27	2	2.94	0.22^ns^	27	–	27	–	–	–
HKI193-2 × HP704-22	BC_1_F_1_	120	80	40	–	1	13.33	0.0003^**^	40	21	19	–	1	0.10	0.75^ns^	19	7	12	1	1.31	0.25^ns^
	BC_2_F_1_	122	108	14	–	1	72.42	0.0001^**^	14	8	6	–	1	0.28	0.59^ns^	6	–	6	–	–	–
	BC_2_F_2_	110	53	26	31	2	39.38	0.0001^**^	110	33	45	32	2	3.66	0.16^ns^	32	–	32	–	–	–
	BC_2_F_3_	142	–	–	142	–	–	–	142	35	67	40	2	0.81	0.67^ns^	40	–	40			

** *Significant at P = 0.01*,

* *Significant at P = 0.05, ns, non-significant; N, No. of plants genotyped; df, degrees of freedom; C^+^, favorable allele of crtRB1; C, unfavorable allele of crtRB1; L^+^, favorable allele of lcyE; L, unfavorable allele of lcyE; o2, favorable allele of opaque2; O2, unfavorable allele of opaque2*.

**Table 5:** Recovery of recipient parent genome (RPG) (%) of the provitamin A improved lines.

**S. No**.	**Cross**	**Generations**	**Range of RPG (%)**
1	HKI161 × HP704-23	BC_1_F_1_	71.49–80.26
		BC_2_F_1_	85.53–88.16
		BC_2_F_2_	88.60–92.98
2	HKI163 × HP704-22	BC_1_F_1_	70.47–75.98
		BC_2_F_1_	83.07–86.22
		BC_2_F_2_	83.86–91.73
3	HKI193-1 × HP704-23	BC_1_F_1_	70.68–80.83
		BC_2_F_1_	85.71–90.60
		BC_2_F_2_	87.97–92.11
4	HKI193-2 × HP704-22	BC_1_F_1_	72.98–80.65
		BC_2_F_1_	85.89–88.31
		BC_2_F_2_	86.69–91.53

### Evaluation of introgressed inbreds

The carotenoid analysis of MABB-derived selected introgressed progenies of HKI161, HKI163, HKI193-1, and HKI193-2 showed a significant increase over their respective recurrent parents (Table 6). The proA concentration among improved inbreds ranged from 7.38 to 13.59 μg/g, compared to 1.65–2.04 μg/g among recurrent parents (Table 6). An average of 6-fold increase in proA was recorded among introgressed progenies. The lysine and tryptophan among the MABB-derived progenies (lysine: 0.274–0.394%, tryptophan: 0.071–0.084%) were *at par* with their respective parental lines (Table 6). The plant phenotypic characteristics and grain yield attributing traits of the introgressed lines were similar to their respective recurrent parents (Table 7). The opaqueness of 25–50% was recorded among introgressed progenies of HKI161 and HKI163, while 50–75% and 95–100% was observed among HKI193-1 and HKI193-2 -based introgressed lines, respectively. The degree of opaqueness is similar to proportion observed among the recurrent parents.

**Table 6:** Biochemical evaluation of selected introgressed progenies with their respective recurrent and donor parents.

**S. No**.	**Genotype**	**proA**	**FC**	**RPG (%)**	**Lysine (%)**	**Tryptophan (%)**
1.	HKI161-24-62-53-38	13.09	6.4	91.23	0.346	0.077
2.	HKI161-24-62-53-61	12.13	5.9	92.54	0.339	0.075
3.	HKI161-24-62-53-67	13.59	6.6	90.35	0.322	0.081
4.	HKI161 (RP)	2.04			0.308	0.076
5.	HKI163-2-90-10-7	7.38	4.5	91.34	0.345	0.080
6.	HKI163-2-90-17-41	7.98	4.8	91.34	0.342	0.075
7.	HKI163-2-90-17-60	9.32	5.6	90.16	0.314	0.084
8.	HKI163 (RP)	1.65			0.347	0.082
9.	HKI193-1-1-8-5-25	10.21	5.6	91.73	0.322	0.081
10.	HKI193-1-1-8-5-38	11.35	6.2	91.35	0.347	0.071
11.	HKI193-1-1-8-5-116	10.50	5.7	92.11	0.274	0.080
12.	HKI193-1 (RP)	1.84			0.323	0.078
13.	HKI193-2-10-8-34-46-10	11.07	6.3	90.32	0.394	0.072
14.	HKI193-2-10-8-34-52-46	12.18	7.0	91.13	0.306	0.079
15.	HKI193-2-10-8-34-68-34	11.37	6.5	91.53	0.366	0.074
16.	HKI193-2 (RP)	1.74			0.304	0.071
17.	HP704-22 (DP)	16.05			0.176	0.028
18.	HP704-23 (DP)	15.28			0.192	0.035

*proA, provitamin A; FC, Fold Change; RP, Recurrent Parents; DP, Donor Parents*.

**Table 7:** Morphological characterization of improved lines with their respective recurrent parents.

**S. No**.	**Genotypes**	**MF**	**FF**	**PH**	**EH**	**EL**	**EW**	**NR**	**NKR**	**TW**
		**(days)**	**(days)**	**(cm)**	**(cm)**	**(cm)**	**(cm)**	**(no.)**	**(no.)**	**(g)**
1.	HKI161-24-62-53-38	53.00	56.00	181.00	72.33	11.47	3.10	12.67	19.00	31.17
2.	HKI161-24-62-53-61	54.00	56.00	185.33	67.33	12.90	3.07	12.00	20.00	31.27
3.	HKI161-24-62-53-67	53.00	56.00	188.33	72.33	12.87	2.93	10.67	17.33	29.80
4.	HKI161 (RP)	53.00	56.00	181.67	69.33	12.07	3.13	12.00	20.67	31.57
5.	HKI163-2-90-10-7	60.00	65.00	170.67	81.00	13.17	3.10	12.67	24.33	27.03
6.	HKI163-2-90-17-41	60.00	64.00	172.00	77.67	11.80	2.67	12.67	23.33	25.37
7.	HKI163-2-90-17-60	61.00	65.00	167.00	76.67	11.53	2.33	11.33	20.00	27.27
8.	HKI163 (RP)	60.00	64.00	173.33	76.00	11.57	2.73	12.00	22.33	27.37
9.	HKI193-1-1-8-5-25	58.00	62.00	168.33	58.33	9.77	2.10	10.67	21.67	20.47
10.	HKI193-1-1-8-5-38	59.00	63.00	161.00	49.33	10.33	2.03	11.33	19.67	18.97
11.	HKI193-1-1-8-5-116	58.00	62.00	159.00	52.67	11.67	2.40	12.00	22.00	19.63
12.	HKI193-1 (RP)	59.00	63.00	161.00	50.00	10.13	2.27	11.33	18.67	19.47
13.	HKI193-2-10-8-34-46-10	56.00	60.00	163.33	71.33	8.83	2.23	10.67	17.33	19.60
14.	HKI193-2-10-8-34-52-46	56.00	60.00	161.33	74.67	9.67	2.40	12.33	22.67	19.40
15.	HKI193-2-10-8-34-68-34	58.00	62.00	168.67	76.67	10.93	2.43	11.33	20.67	16.63
16.	HKI193-2 (RP)	56.00	59.00	166.67	70.00	10.17	2.43	12.67	20.33	18.23
	SE	0.69	0.83	2.30	2.56	0.32	0.09	0.18	0.50	1.34

*MF, days to 50% male flowering; FF, days to 50% female flowering; PH, plant height; EH, ear height; EL, ear length; EW, ear width; NR, number of rows; NKR, number of kernels per row; TW, 100-seed weight; SE, Standard Error*.

### Evaluation of reconstituted hybrids

**Figure 3:**
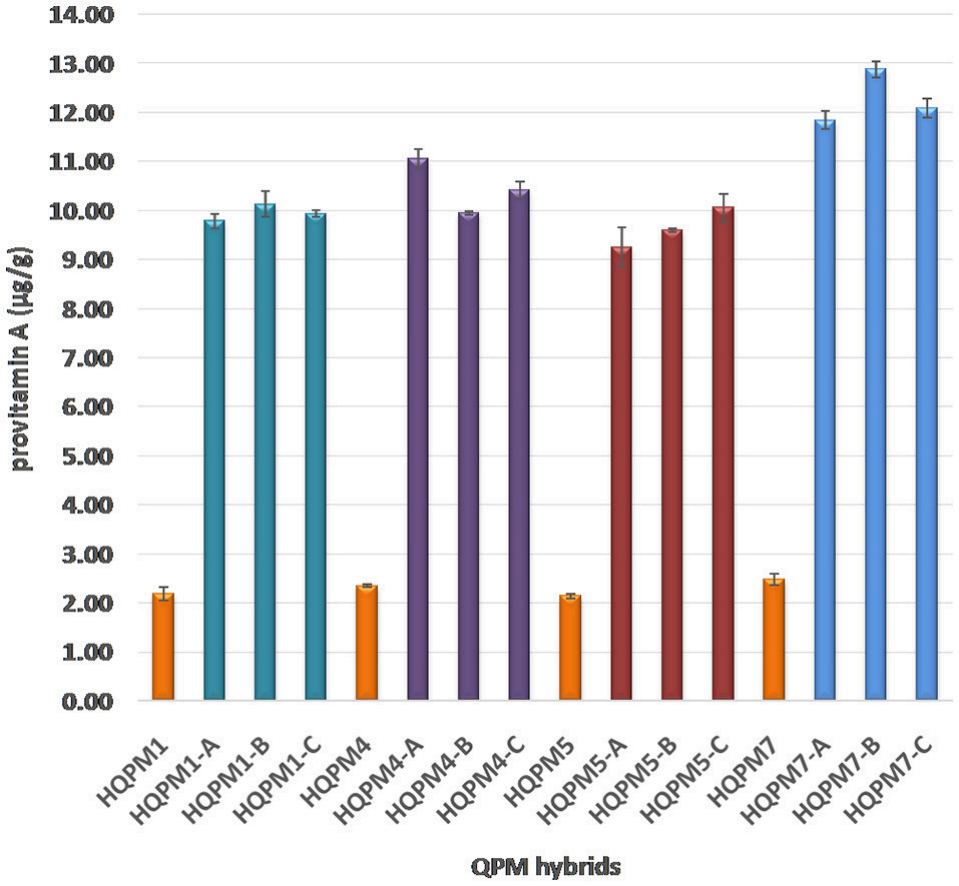
Provitamin A concentration in original- and reconstituted- hybrids.

**Figure 4:**
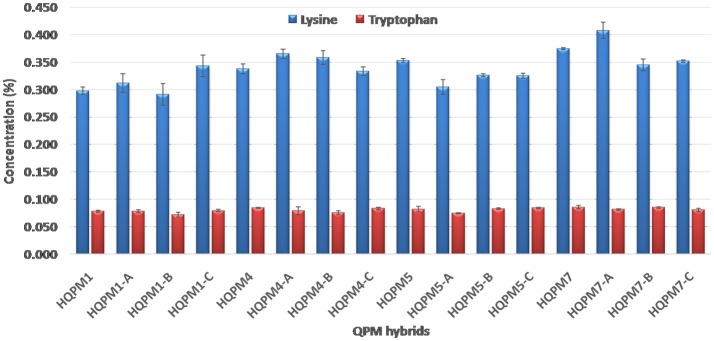
Lysine and tryptophan concentration in original- and reconstituted- hybrids.

The proA among reconstituted hybrids showed an average of 4.5-folds increase over their original versions. The proA of the newly derived hybrids ranged from 9.25 to 12.88 μg/g compared to 2.14–2.48 μg/g among the original hybrids (Figure 3). The mean proA in HQPM1-, HQPM4-, HQPM5-, and HQPM7-based reconstituted hybrids was 9.95, 10.47, 9.63, and 12.27 μg/g, respectively (Figure 3). The proA fold change was as high as 4.6 times in HQPM1-B over its original hybrid, while HQPM4-A, HQPM5-C, and HQPM7-B had 4.7-, 4.7-, and 5.1-fold increase in proA over their respective original hybrids. The lysine and tryptophan among the MABB-derived versions was *at par* with their respective original versions of hybrid (Figure 4). Among reconstituted hybrids, lysine ranged from 0.291 to 0.365%, while tryptophan varied from 0.072 to 0.085%. The improved hybrids showed high degree of resemblance for agronomic traits with their respective original hybrids across locations (Table 8 and Table S4). The grain yield and attributing traits of MABB-derived hybrids were also *at par* with their respective original versions (Table 8, Table S4).

**Table 8:** Combined analysis of reconstituted hybrids along with original hybrids across the locations.

**S. No**.	**Genotypes**	**MF**	**FF**	**PH**	**EH**	**EL**	**EW**	**NR**	**NKR**	**TW**	**GY**
		**(days)**	**(days)**	**(cm)**	**(cm)**	**(cm)**	**(cm)**	**(no.)**	**(no.)**	**(g)**	**(kg/ha)**
1	HQPM1	59.00	60.50	180.75	81.90	15.10	3.65	14.30	28.00	25.25	6559.00
2	HQPM1-A	57.00	59.75	180.30	79.40	15.60	3.80	14.00	30.30	26.90	6770.00
3	HQPM1-B	57.50	59.50	185.95	80.65	15.50	3.60	14.50	28.20	27.40	6843.00
4	HQPM1-C	58.50	60.50	174.70	76.55	15.30	3.90	14.40	30.00	26.40	6917.50
5	HQPM4	60.00	60.75	197.20	86.25	17.10	4.05	14.05	33.35	30.20	7315.00
6	HQPM4-A	58.75	58.75	195.00	82.50	16.90	4.00	14.30	32.05	29.30	7534.00
7	HQPM4-B	57.50	59.25	199.40	86.25	17.45	3.95	14.50	32.75	29.20	7239.00
8	HQPM4-C	58.00	57.75	189.60	81.75	16.90	4.00	14.15	31.50	30.20	7468.50
9	HQPM5	58.25	59.50	180.95	76.25	15.40	3.70	14.70	28.15	27.85	7793.50
10	HQPM5-A	56.50	57.25	197.20	83.80	15.75	3.95	14.85	30.90	28.90	7606.50
11	HQPM5-B	58.25	59.00	186.25	84.70	15.65	4.00	14.00	30.90	27.10	7338.00
12	HQPM5-C	58.50	59.50	191.05	84.05	15.50	4.15	15.35	29.25	26.65	7728.00
13	HQPM7	59.25	59.75	187.60	76.60	16.20	3.95	14.35	29.50	29.80	7513.50
14	HQPM7-A	58.50	59.50	188.75	80.00	16.40	4.00	14.35	31.25	29.30	7632.00
15	HQPM7-B	59.25	60.25	194.05	85.30	16.70	4.05	14.50	30.80	30.45	7415.50
16	HQPM7-C	57.25	58.50	183.90	80.20	16.75	3.90	14.60	31.10	27.65	7352.50
	SE	0.96	1.32	5.31	4.24	0.50	0.16	0.49	0.89	0.78	644.67

*MF, days to 50% male flowering; FF, days to 50% female flowering; PH, plant height; EH, ear height; EL, ear length; EW, ear width; NR, number of rows; NKR, number of kernels per row; TW, 100-seed weight; GY, grain yield; DL, Delhi; DW, Dharwad; HQPM1-A, HKI193-1-1-8-5-38 × HKI163-2-90-17-60; HQPM1-B, HKI193-1-1-8-5-116 × HKI163-2-90-10-7; HQPM1-C, HKI193-1-1-8-5-25 × HKI163-2-90-17-41; HQPM4-A, HKI193-2-10-8-34-68-34 × HKI161-24-62-53-61; HQPM4-B, HKI193-2-10-8-34-52-46 × HKI161-24-62-53-38; HQPM4-C, HKI193-2-10-8-34-52-46 × HKI161-24-62-53-61; HQPM5-A, HKI163-2-90-10-7 × HKI161-24-62-53-61; HQPM5-B, HKI163-2-90-10-7 × HKI161-24-62-53-67; HQPM5-C, HKI163-2-90-17-41 × HKI161-24-62-53-67; HQPM7-A, HKI193-1-1-8-5-116 × HKI161-24-62-53-61; HQPM7-B, HKI193-1-1-8-5-25 × HKI161-24-62-53-38; HQPM7-C, HKI193-1-1-8-5-38 × HKI161-24-62-53-61; SE, Standard Error*.

## Discussion

Normal maize endosperm contains low lysine and tryptophan, however their level is elevated by almost double in QPM genotypes due to recessive *o2* present on chromosome 7 (Mertz et al., 1964; Vasal, 2000). However QPM like traditional normal maize genotypes also possesses very low proA carotenoids (Gupta et al., 2015). Animal's metabolism cannot synthesize lysine, tryptophan, and proA in their body, therefore the requirement is to be met from food sources (Pixley et al., 2013). Mutant version of *crtRB1* and *lcyE* enhances proA level and makes maize grain more nutritious for human/ animal consumption (Harjes et al., 2008; Yan et al., 2010; Babu et al., 2013). The present study used MABB for combining the favorable alleles of *crtRB1, lcyE*, and *o2* into four elite QPM inbreds, viz., HKI161, HKI163, HKI193-1, and HKI193-2 to develop biofortified maize hybrids rich in proA, lysine and tryptophan. In the present study, distinct variation for target traits revealed through marker polymorphism for *crtRB1* (Muthusamy et al., 2014; Liu et al., 2015), *lcyE*, and *o2* (Gupta et al., 2013; Hossain et al., 2017) was observed between recurrent and donor parents

Severe segregation distortion (SD) was realized for *crtRB1* across the generations and crosses (Babu et al., 2013; Muthusamy et al., 2014; Liu et al., 2015). This SD is possibly due to the presence of gametophytic factors, mutants like defective kernel, male sterility and embryo-specific mutation (Neuffer et al., 1997; Lu et al., 2002). SD thus necessitates assaying of large population for achieving sufficient foreground positive genotypes in MABB programme. Conversely, *lcyE* gene did not show SD. In majority of populations, *o2* also segregated as per Mendelian inheritance (Gupta et al., 2013; Hossain et al., 2017) with an exception in one population (Jompuk et al., 2011). Marker-assisted background selection using SSRs helped to achieve high recovery of RPG in just two backcross generations (Gupta et al., 2013; Muthusamy et al., 2014; Feng et al., 2015; Liu et al., 2015; Hossain et al., 2017). The introgressed progenies with high RPG showed high degree of resemblance with their corresponding recurrent parent for plant architecture and ear- and grain- characteristics (Gupta et al., 2013; Choudhary et al., 2014; Muthusamy et al., 2014). This high degree of phenotypic similarity among the reconstituted hybrids is also attributed to high RPG of the parental inbreds (Gupta et al., 2013; Muthusamy et al., 2014; Hossain et al., 2017).

Introgressed inbreds possessed 5–7-folds more proA than their respective recipient parents, while the reconstituted hybrids had 4–5-folds higher proA over their original versions. Expression analysis revealed that mutant *crtRB1*-transcripts was drastically reduced, leading to lesser amount of β*-hydroxylase* and lesser conversion of β-carotene to further components (Vallabhaneni et al., 2009; Yan et al., 2010). Similarly, reduced transcript level of *lcyE*-mutant produces lesser concentration of ε*-cyclase* compared to wild type allele, thereby shifting more lycopene flux from α-branch to β-branch of the carotenoid biosynthesis pathway (Harjes et al., 2008). The difference in expression levels of *crtRB1* and *lcyE* genes was significant in endosperm, but not in embryos and leaves (Babu et al., 2013). The cumulative advantage of *crtRB1* and *lcyE* for proA over individual effects has been reported in various studies (Azmach et al., 2013; Gebremeskel et al., 2017; Zunjare et al., 2017b). However, the favorable alleles of the both genes (*crtRB1* and *lcyE*) occur in low frequency in the maize germplasm (Azmach et al., 2013; Babu et al., 2013; Muthusamy et al., 2015; Gebremeskel et al., 2017). Even in association mapping panel used by Harjes et al. (2008) and Yan et al. (2010) did not find any genotypes with favorable allele of both the genes (*crtRB1* and *lcyE*).

The range of proA concentration was observed among both MABB-derived inbreds and hybrids, despite having the same allele of *crtRB1* and *lcyE*. This variation is possibly due to varied interaction of *crtRB1* and *lcyE* with the genome (Babu et al., 2013; Muthusamy et al., 2014). Also, improved progenies of four crosses revealed kernel proA concentration lower than their respective donor parents (Table 2). This suggests that other genetic loci or QTLs apart from favorable alleles of the *crtRB1* and *lcyE* genes, contribute to the accumulation of proA (Wong et al., 2004; Chander et al., 2008; Zhou et al., 2012; Kandianis et al., 2013). Current study has achieved 70% of target level 15 μg/g proA in reconstituted hybrids (mean: 10.58 μg/g) which emphasize the need for further introgression of genetic loci like *crtRB3, CCD1*, and *ZEP1* (Zhou et al., 2012; Suwarno et al., 2015).

The nutritional benefit of QPM with enhanced lysine and tryptophan were also conserved in the MABB-derived lines and their reconstituted hybrids. The *o2* leads to reduction of zein proteins, with a concurrent increase in non-zein proteins rich in lysine and tryptophan (Ueda et al., 1992). *o2* also down regulates the synthesis of *lysine ketoglutarate reductase* resulting in increased levels of free lysine (Kemper et al., 1999). Besides, it is also involved in regulation of various lysine-rich proteins and enzymes (Jia et al., 2013). The variation of lysine and tryptophan observed in the *o2*-based introgressed progenies is due to various modifier loci including *opaque16* that affect regulation of amino acid biosynthesis (Wu et al., 2002; Yang et al., 2005; Pandey et al., 2015; Sarika et al., 2017). Similarly, the variation for lysine and tryptophan among *o2*-introgressed progenies was also observed by Gupta et al. (2013) and Hossain et al. (2017) in their MABB programmes.

The grain yield of reconstituted hybrids was also *at par* with the original hybrids. The similarity was due to indirect selection of loci for yield potential and various agronomic traits through background selection. Yield has not been used as the criterion of selecting the segregants, however the introgressed progenies led to the development of heterotic hybrids which were similar to the original hybrids (Gupta et al., 2013; Muthusamy et al., 2014; Hossain et al., 2017). The study thus implemented a successful demonstration of MABB augmented with stringent phenotypic selection for agro-morphological characters. The present investigation was the first report of combining favorable alleles of *crtRB1, lcyE*, and *o2* in a single genetic background. During the year 2017, *o2*, and *crtRB1*-based “Pusa Vivek QPM9 Improved” maize hybrid with high proA, lysine and tryptophan has been released by ICAR in India (Muthusamy et al., 2014). Pusa Vivek QPM9 Improved provides an average grain yield of 5,588 and 5,916 kg/ha in Zone-I and Zone-IV, respectively (Annual Progress Report *Kharif* Maize, 2016). In comparison, the newly developed proA rich QPM hybrids in the present study possessed higher average grain yield (mean: 7,314 kg/ha). Moreover, these hybrids (Zone-II, III, IV, and V, Table 1) are also adapted to diverse agro-ecological zones.

The improved inbreds thus developed here can be used as donor lines for simultaneous introgression of *o2, crtRB1*, and *lcyE* in the breeding programme. Further, the improved inbreds can be crossed among them to generate different F_2_ populations, where from new inbreds with high proA, lysine and tryptophan can be derived using pedigree method. The nutritionally improved hybrids can be grown for cultivation for commercial usage of biofortified grains as food and feed. The significance of biofortified maize for human health has very well observed in many countries (Bouis and Saltzman, 2017). The benefit of QPM in human health and poultry birds is also well documented (Gunaratna et al., 2010; Panda et al., 2014). Biofortified orange maize was found to be as efficacious as a vitamin A supplement in children (Gannon et al., 2014). Dubey et al. (2017) using caco-2 cell model demonstrated that proA rich maize hybrids having *crtRB1* allele possessed enhanced bioavailability of β-carotene. Chickens fed with biofortifid maize produced eggs rich in proA (Liu et al., 2012; Heying et al., 2014; Moreno et al., 2016; Sowa et al., 2017). A study further revealed that proA biofortified fed chickens had higher redness and yellowness and lower lightness in the meat and skin color than white maize fed chickens (Odunitan-Wayas et al., 2016). Thus, both direct consumption through foods and indirect consumption through chicken -eggs and -meats, proA rich maize contributes to nutritional security. Lividini and Fiedler (2015) demonstrated the great promise of proA rich maize for becoming a highly cost-effective strategy for reducing malnutrition. Biofortified high yielding maize hybrid rich in proA, lysine and tryptophan nutrients would be a sustainable delivery tools to overcome micronutrient malnutrition.

## Conclusions

We report here the development of four maize hybrids using marker-assisted stacking of *o2, crtRB1*, and *lcyE*. The hybrids were evaluated at two locations and provided similar grain yield potential of the original hybrids. The inbreds with elevated lysine, tryptophan and proA concentration can be used as potential donors for development of nutrient rich maize cultivars in future breeding programmes. The biofortified maize hybrids enriched with proA, lysine and tryptophan possess great potential to simultaneously alleviate vitamin A deficiency and protein-energy malnutrition across the world.

## Author contributions

Conduct of all experiments: RZ; Development of segregating progenies: FH and VM; Morphological characterization: FH and JB; Phenotyping for kernel quality: VM, AB and SS; Analysis on kernel modification: HC; Statistical analyses: FH and NT; Drafting of the manuscript: RZ and FH; Designing of the experiment: FH and HG.

### Conflict of interest statement

The authors declare that the research was conducted in the absence of any commercial or financial relationships that could be construed as a potential conflict of interest.

## Supplementary material

Figure S1 Foreground selection for *crtRB1, lcyE*, and *o2* in BC_1_F_1_ generation. Star indicates plants heterozygous for *crtRB1/lcyE* and homozygous for *o2* allele. RP, Recurrent Parent; DP, Donor Parent.

Table S1 Details of genetic materials used in MABB.

Table S2 Details of populations generated under MABB.

Table S3 List of SSR markers used in background selection.

Table S4 Morphological characterization of reconstituted hybrids along with their original versions.
