# Supplementary figures and images for: Development of Biofortified Maize Hybrids through Marker-Assisted Stacking of β-Carotene Hydroxylase, Lycopene-ε-Cyclase and Opaque2 Genes
Source: Front Plant Sci. 2018 Feb 20;9:178. doi: 10.3389/fpls.2018.00178 (PMC5826225; doi:10.3389/fpls.2018.00178)

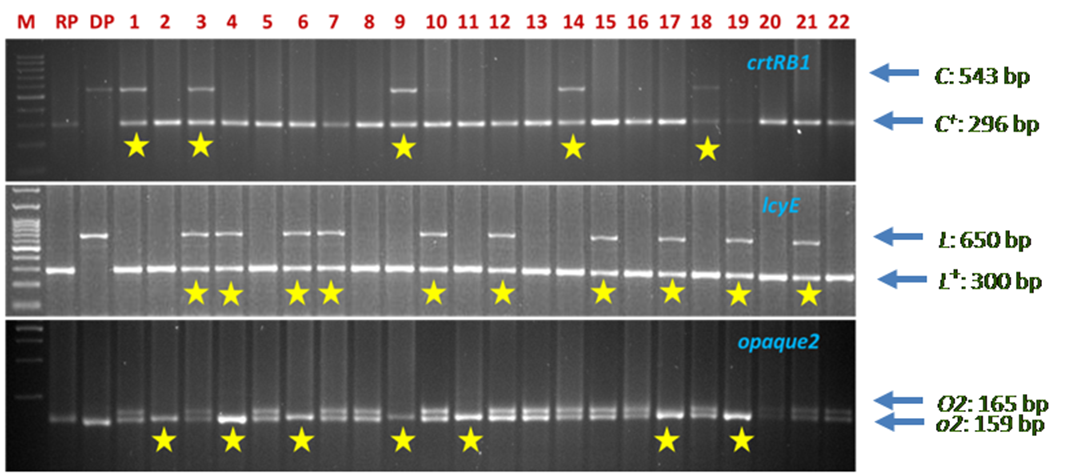

Supplement: Figure S1 — Foreground selection for crtRB1, lcyE, and o2 in BC1F1 generation. Star indicates plants heterozygous for crtRB1/lcyE and homozygous for o2 allele. RP, Recurrent Parent; DP, Donor Parent. [file Image1.TIF]
