# Supplementary material for: Development of Biofortified Maize Hybrids through Marker-Assisted Stacking of β-Carotene Hydroxylase, Lycopene-ε-Cyclase and Opaque2 Genes
Source: Front Plant Sci. 2018 Feb 20;9:178. doi: 10.3389/fpls.2018.00178 (PMC5826225; doi:10.3389/fpls.2018.00178)
Supplement: Table S1 — Details of genetic materials used in MABB. [file Table1.DOC]

Table S1. Details of genetic materials used in MABB

| **S. No.** | **Genotypes** | **Pedigree** | **Kernel colour** | **Maturity (days)** | **Accession number** | **Institution** |
| --- | --- | --- | --- | --- | --- | --- |
| **Recurrent parents** | | | |  |  |  |
| 1. | HKI161 | Selection from CML161 (P25QPM) | Orange | 94-96 | IC553428 | CCSHAU, Uchani |
| 2. | HKI163 | Selection from CML163 (P26QPM) | Yellow | 95-98 | IC470150 | CCSHAU, Uchani |
| 3. | HKI193-1 | Selection from CML193 | Yellow | 92-95 | IC470149 | CCSHAU, Uchani |
| 4. | HKI193-2 | Selection from CML193 | Yellow | 96-100 | IC584588 | CCSHAU, Uchani |
| **Donor parents** | | | |  |  |  |
| 1. | HP704-22 | (KUI carotenoid syn-FS11-1-1-B-B-B/(KU1409/DE3/KU1409)S2-18-2-B)-B-3(MAS:L4H1)-1-B-B-B | Yellow | 97-103 | EC737637-737641 | CIMMYT, Mexico |
| 2. | HP704-23 | (KUI carotenoid syn-FS17-3-2-B-B-B/(KU1409/DE3/KU1409)S2-18-2-B)-B-1(MAS:L4H1)-1-B-B-B | Yellow | 98-101 | EC737637-737642 | CIMMYT, Mexico |
