# Supplementary material for: Development of Biofortified Maize Hybrids through Marker-Assisted Stacking of β-Carotene Hydroxylase, Lycopene-ε-Cyclase and Opaque2 Genes
Source: Front Plant Sci. 2018 Feb 20;9:178. doi: 10.3389/fpls.2018.00178 (PMC5826225; doi:10.3389/fpls.2018.00178)
Supplement: Table S2 — Details of populations generated under MABB. [file Table2.DOC]

Table S2. Details of populations generated under MABB

| **S. No.** | **Generations** | **Seasons** | **Location** |
| --- | --- | --- | --- |
| 1. | P_1_ and P_2_ | *rainy 2012* | IARI Experimental Farm, New Delhi |
| 2. | F_1_ | *winter* 2012-13 | Winter Nursery Centre, Hyderabad |
| 3. | BC_1_F_1_ | *rainy 2013* | IARI Experimental Farm, New Delhi |
| 4. | BC_2_F_1_ | *winter* 2013-14 | Winter Nursery Centre, Hyderabad |
| 5. | BC_2_F_2_ (BC_2_F_2_-I) | *rainy* 2014 | IARI Experimental Farm, New Delhi |
| 6. | BC_2_F_3_ (BC_2_F_3_-I/ BC_2_F_2_-II) | *winter* 2014-15 | Winter Nursery Centre, Hyderabad |
| 7. | BC_2_F_4_ (BC_2_F_4_-I/ BC_2_F_3_-II) | *rainy* 2015 | IARI Experimental Farm, New Delhi |
| 8. | Generation of F_1_ hybrids | *winter* 2015-16 | Winter Nursery Centre, Hyderabad |
| 9. | Evaluation of reconstituted hybrids | *rainy* 2016 | IARI Experimental Farm, New Delhi &  IARI Regional Research Centre, Dharwad |

Populations in parenthesis is specific to conversion of HKI163
