# Supplementary material for: Development of Biofortified Maize Hybrids through Marker-Assisted Stacking of β-Carotene Hydroxylase, Lycopene-ε-Cyclase and Opaque2 Genes
Source: Front Plant Sci. 2018 Feb 20;9:178. doi: 10.3389/fpls.2018.00178 (PMC5826225; doi:10.3389/fpls.2018.00178)
Supplement: Table S3 — List of SSR markers used in background selection. [file Table3.DOC]

Table S3. List of SSR markers used in background selection.

| **S. No.** | **Primer** | **Repeat** | **Bin** | **Forward base (5' to 3')** | **Reverse base (5' to 3')** |
| --- | --- | --- | --- | --- | --- |
| 1 | *phi056* | CCG | 1.00 | ACTTGCTTGCCTGCCGTTAC | CGCACACCACTTCCCAGAA |
| 2 | *bnlg147* | NA | 1.02 | AGGAAGCTTTGGTCAAGTCTTA | GCTCACTCGATTTGTTGTGCTA |
| 3 | *bnlg1203* | (AG)17 | 1.03 | GACCCGTCTCTCTTGAGTGC | GTCTGTCTGCACCCGTTTTT |
| 4 | *bnlg439* | NA | 1.03 | TTGACATCGCCATCTTGGTGACCA | TCTTAATGCGATCGTACGAAGTTGTGGAA |
| 5 | *bnlg2204* | (AG)24 | 1.03 | AGGCGACTTAGCTGCAGAAG | CGACTTTCGGTTTGGAAAAG |
| 6 | *bnlg652* | NA | 1.05 | CGCACGTCGGGAGAGAGGGAGA | GCCGCAAACATAGCCGCCAAAAAT |
| 7 | *bnlg421* | NA | 1.06 | GGGGCAAGGACTTGTCGGT | AGCCAGTTGCCCAGCATCT |
| 8 | *umc1035* | (CT)19 | 1.06 | CTGGCATGATCACGCTATGTATG | TAACATCAGCAGGTTTGCTCATTC |
| 9 | *bnlg1057* | (AG)17 | 1.06 | TTCACCGCCTCACATGAC | GCAACGCTAGCTAGCTTTG |
| 10 | *umc1278* | (CT)12 | 1.07 | TGCCATAGTACATGTCCGTCATTC | GTCGGAGGATGATCCCCTATCTAT |
| 11 | *umc1446* | (TAA)7 | 1.08 | GCGCTGCTGCTTCTTAAATTATCT | GATGAGACCACCTACAAGTTCGCT |
| 12 | *bnlg400* | NA | 1.09 | AGCTGTGACTGTGAAGGGAAAA | CGTCACACCGCTGTTTCTTG |
| 13 | *phi064* | ATCC | 1.11 | CCGAATTGAAATAGCTGCGAGAACCT | ACAATGAACGGTGGTTATCAACACGC |
| 14 | *bnlg131* | NA | 1.11 | CTCTGCGCTACCTTTCTGAGTC | GCGGAATCCTTGTGTTCTTG |
| 15 | *bnlg1297* | (AG)32 | 2.02 | TCTCGATCGCTCCGATCTAT | GACTCAACTCCAAAAGGCGA |
| 16 | *bnlg1613* | (AG)27 | 2.04 | GGGGATGATTCCGATAGGC | GCGCTCTCTTTTCCCTCTCT |
| 17 | *phi083* | AGCT | 2.04 | CAAACATCAGCCAGAGACAAGGAC | ATTCATCGACGCGTCACAGTCTACT |
| 18 | *bnlg371* | NA | 2.05 | CAACGCGAAGCAGAGATAAAA | TCGTCGCATGACCATAGTAGC |
| 19 | *bnlg1225* | (AG)14 | 2.06 | GCAGTAGAAGAGCGAGCGAG | CATACGCTGTCACTGCCACT |
| 20 | *bnlg1396* | (AG)15 | 2.06 | CGCATTTCTCCTGCAGTACA | TGCTTGAGTCGTCGAATCTG |
| 21 | *bnlg1045* | (AG)23 | 2.07 | TCCCCGATAGCATATCGATC | GTGACTTTGGGGAGTTTGGA |
| 22 | *umc1497* | (GAC)4 | 2.07 | GAGGAAGACGGAGACGGCAC | TAGACGAGATCCTCTGAGGCAATG |
| 23 | *umc2129* | (CGC)5 | 2.07 | ACGTGGTCATCACTCACCGC | AAGGAGGAGCGTTCTCGTGG |
| 24 | *bnlg1316* | (AG)13 | 2.08 | CGAAACAGAGCCCAAAAGAC | GATCCGCGTCTAGCCCCT |
| 25 | *bnlg1140* | (AG)35 | 2.08 | TAGGCCATATTGGCCCATG | AATGCCGTGGACGTAAGATC |
| 26 | *phi427434* | ACC | 2.08 | CAACTGACGCTGATGGATG | TTGCGGTGTTAAGCAATTCTCC |
| 27 | *phi090* | ATATC | 2.08 | CGTGCAAATAATTCCCCGTGGGA | CTACCTATCCAAGCGATGGGGA |
| 28 | *umc2077* | (AGC)4 | 2.09 | AAACTCACTGAACATGATCCTGGC | CTGGTTCGGATGCAAGTAGTCAG |
| 29 | *umc1256* | (CAT)5 | 2.09 | CATCTCGACCTTTGACATTCTCCT | AGAAGACGATGATGATGATGCAGA |
| 30 | *bnlg1520* | (AG)22 | 2.09 | ACAGCTGCGTAGCTTCTTCC | TCCTCTTGCTCTCCATGTCC |
| 31 | *umc2101* | (AG)7 | 3.00 | CCCGGCTAGAGCTATAAAGCAAGT | CTAGCTAGTTTGGTGCGTGGTGAT |
| 32 | *phi404206* | NA | 3.01 | GTACCCGCAACAAATGCC | CGTCTCATGAAGCTTTCTTGG |
| 33 | *phi374116* | NA | 3.02 | NA | NA |
| 34 | *phi193225* | AAC | 3.02 | GCTCTTGGCGTGCTTCTT | GCGGGGAGGTGAAGAGCTA |
| 35 | *bnlg1628* | (AG)16 | 3.04 | GTAGGGTTCAAGGAGGCACA | CTCTCTGGTGAGCTGGCTTT |
| 36 | *phi099* | AC | 3.04 | TACAAAAATCAGGACTGCGAAAAACCCAA | GTCGGTGTGTGATCCTTCCAC |
| 37 | *bmc2136* | (CA)31 | 3.04 | TGCTCCTTCTCGAGCACC | ATGGACGTACGGCAGACTCT |
| 38 | *phi029* | AG | 3.04 | TTGTCTTTCTTCCTCCACAAGCAGCGAA | ATTTCCAGTTGCCACCGACGAAGAACTT |
| 39 | *bnlg1638* | (AG)25 | 3.04 | CATATCTCTAGCTTCTCGTCTTCG | ACACCGATCGAGGAAGAATG |
| 40 | *bnlg420* | NA | 3.05 | CTTGCGCTCTCCTCCCCTT | GGCCAGCTCACTGCTCACT |
| 41 | *phi053* | ATAC | 3.05 | AACCCAACGTACTCCGGCAG | CTGCCTCTCAGATTCAGAGATTGAC |
| 42 | *umc1102* | GGAT | 3.05 | AAAATTAAAGTCAAGAGCGGGGAG | TCTAGCCGTAGCTTTAGCTGCATT |
| 43 | *bnlg1035* | (AG)13 | 3.05 | TGCTTGCACTGTCAGGAATC | CAGCTCTGACACACCACACA |
| 44 | *phi073* | ATAC | 3.05 | GTGCGAGAGGCTTGACCAA | AAGGGTTGAGGGCGAGGAA |
| 45 | *bnlg1505* | (AG)27 | 3.05 | GAAAGACAAGGCGAAGTTGG | GCTTCTGAACTGGATCGGAG |
| 46 | *bnlg1951* | (AG)11 | 3.06 | CAAGCGTTCTGGTTTTCACA | ATTGCCGTTCTCAAAACGAG |
| 47 | *umc1311* | (TCTT)4 | 3.06 | CTCGACAACTTTTGGCTTCGTACT | AAAGCGATGCCATCAGAATCTAAG |
| 48 | *umc1489* | (GCG)5 | 3.07 | TTAATAGCTACCCGCAACCAAGAA | CTGAGCCACAGTACCTTGCTGTT |
| 49 | *umc1690* | (GCA)4 | 3.07 | ACCTTAGTTACACAGGCACACGGT | GGTGATGGGATTTTCGCATTATTA |
| 50 | *umc1915* | (ACA)6 | 3.08 | AGACGAGTTAAACCTCCATCATGC | CCTACCCCAACTTGCTTGAGACTA |
| 51 | *phi088* | ACT | 3.08 | CTTCTGTTCCGCCATCCAGTATGT | GATTGCGATAAGCATTGCGGCAGTT |
| 52 | *umc1320* | (GAAC)4 | 3.08 | TGCGAAATCTGTATACCATAGGCA | CTCTTTTAGCAGTGTGCCGAATTT |
| 53 | *umc2174* | (CGA)4 | 3.08 | GTACGTACGCAGCCACTTGTCAG | ACATAAATAAAACGTGTGCCGCAG |
| 54 | *umc2152* | (TG)8 | 3.09 | TAGCTTCACCTGATGATCTTGCAC | CCTTTGTCTTCCGCTATCTTCCTT |
| 55 | *bnlg372* | NA | 4.00 | TTCACATGCCATCCTCCTATAT | TATCCCTCTCTGATCACGTTGG |
| 56 | *phi021* | AG | 4.03 | TTCCATTCTCGTGTTCTTGGAGTGGTCCA | CTTGATCACCTTTCCTGCTGTCGCCA |
| 57 | *bnlg1162* | (AG)21 | 4.03 | CATAGCAACAAGGACCCTACG | CGTCCTAGTGGAACCAGGAA |
| 58 | *phi109188* | AAAG | 4.04 | AAGCTCAGAAGCCGGAGC | GGTCATCAAGCTCTCTGATCG |
| 59 | *phi074* | CAA | 4.04 | CCCAATTGCAACAACAATCCTTGGCA | GTGGCTCAGTGATGGCAGAAACT |
| 60 | *phi079* | AGATG | 4.05 | TGGTGCTCGTTGCCAAATCTACGA | GCAGTGGTGGTTTCGAACAGACAA |
| 61 | *umc1303* | (CCG)4 | 4.05 | ATCCTAGGAAAGCAGGGAGGG | CTTGGTAGCTTCGTATTCGACGAG |
| 62 | *bnlg1729* | (AG)21 | 4.05 | CCAATCCCGACAGGTTCTGT | GACATCTGTGCCCAACAATG |
| 63 | *umc1869* | (GGT)6 | 4.06 | CGAGCGCTCTAGACACGATTTT | GAACTGGAGGAGCGAGCATGTAT |
| 64 | *bnlg1137* | (AG)34 | 4.06 | ATGAGCTCAGTCACACTGTAGTG | ACTGATGACTGGTCCATGCA |
| 65 | *bnlg1621* | (AG)18 | 4.06 | CTCTTCGATCTTTAAGAGAGAGAGAG | ACACGAGGCACTGGTACTAACG |
| 66 | *bnlg252* | NA | 4.06 | CGTTCTCCGTACAGCACAGACCAACGT | CTCAGATGAACTCCTCAGCAGCTGTAGCCT |
| 67 | *umc2038* | (GAC)4 | 4.07 | ACAGAAACCAATGCATGTGATGAG | TGCATGGTTGCTTCAGCAGT |
| 68 | *phi093* | AGCT | 4.08 | AGTGCGTCAGCTTCATCGCCTACAAG | AGGCCATGCATGCTTGCAACAATGGATACA |
| 69 | *umc2384* | (GCC)5 | 4.08 | CTCTGGACTCGGTTCCTCCTTAC | CTCCTCTTTCCTGATCCTCTGCT |
| 70 | *phi314704* | NA | 4.09 | CCCGATCTACCAGCTTCG | GGTTATTACCGCCATCCAGC |
| 71 | *bnlg1917* | (AG)26 | 4.10 | ACCGGAACAGACGAGCTCTA | TTTGCTTCCAACTCACATGC |
| 72 | *umc2044* | (CGG)6 | 4.10 | ACCTCTCTCCGCCGAAGAATC | CATATAGGTCATGCCTGCCATCTC |
| 73 | *phi076* | AGCGGG | 4.11 | TTCTTCCGCGGCTTCAATTTGACC | GCATCAGGACCCGCAGAGTC |
| 74 | *nc130* | AGC | 5.00 | GCACATGAAGATCCTGCTGA | TGTGGATGACGGTGATGC |
| 75 | *umc1478* | (GGAG)4 | 5.01 | GAAGCTTCTCCTCTCGCGTCTC | CAGTCCCAGACCCTAGCTCAGTC |
| 76 | *bnlg143* | NA | 5.01 | GCACTGCCGGAGTGCCTTCT | ATGCCGTGATCTGTGACATCTAACC |
| 77 | *phi024* | CCT | 5.01 | ACTGTTCCACCAAACCAAGCCGAGA | AGTAGGGGTTGGGGATCTCCTCC |
| 78 | *bnlg1046* | (AG)39 | 5.03 | TGAGCCGAAGCTAACCTCTC | GATGCAAAGGAGGTTCAGGA |
| 79 | *phi096* | AGGTG | 5.03 | TCCACCATTTGACACTTAGGCA | GCGTAGGACGACCGTTGAA |
| 80 | *umc2167* | (CGC)6 | 5.03 | AGACCGGCTAGCGCCATATC | AGTGGTGGACGAAGAGACAAGC |
| 81 | *bnlg653* | NA | 5.04 | CGCATTGCCATGGATGAAGAACTGG | GCAAGCGCCTCACAAGGTATGCACA |
| 82 | *bnlg603* | NA | 5.04 | CTGAGCTGGCCCCTGTGAATGGTG | CGCCCTCCGCTGCGCTTCTCT |
| 83 | *umc2111* | (CTCA)4 | 5.05 | CACGCAACCCACTCATCACTC | CTCACCGCTCTGCTCTGCTATC |
| 84 | *bnlg1847* | (AG)12 | 5.06 | GACGCTAGAGAGAGGCGAAG | ATGTAACAAGAAGGCCCGTG |
| 85 | *bnlg1346* | (AG)24 | 5.07 | CATCATGAAGCAATGAAGCC | CCGCGCCATTATCTAGTTGT |
| 86 | *umc2201* | (GCG)5 | 5.07 | AGGGAAGGGGAAAAGCAGTTAAG | TAGAACGGCGAACAGAAGCAG |
| 87 | *phi330587* | NA | 5.07 | NA | NA |
| 88 | *bnlg386* | NA | 5.09 | CACCCTCCCTTTGCAGGTA | TGGTTTATCAGATAACGATTCAGC |
| 89 | *phi423796* | AGATG | 6.01 | CACTACTCGATCTGAACCACCA | CGCTCTGTGAATTTGCTAGCTC |
| 90 | *umc1186* | (GCT)5 | 6.01 | TCAAGAACATAATAGGAGGCCCAC | AGCCAGCTTGATCTTTAGCATTTG |
| 91 | *phi077* | AG | 6.01 | GAGAAGAGGATCAGGTTCGTTCCA | CGCGTTGTACATCTTGCCTGCTT |
| 92 | *phi389203* | AGC | 6.03 | GACGAAAAGGTGGCTCGT | TGCAGTCCTAGATCAGTTCCAA |
| 93 | *bnlg480* | NA | 6.04 | GACATTTCCAATGGCGGCTTTCC | TCTAGTTATTCCAAGCCCTGGGC |
| 94 | *phi031* | GTAC | 6.04 | GCAACAGGTTACATGAGCTGACGA | CCAGCGTGCTGTTCCAGTAGTT |
| 95 | *umc1857* | (TAA)6 | 6.04 | TTCCTTGCCAACAAATACAAGGAT | GTTCATTGCTTCATCTTGGAACCT |
| 96 | *phi025* | NA | 6.05 | GCAACATCCTGGAGAGCCACTACAAGG | ACAGCCTGTTTTCCTGGACAGTGAACTC |
| 97 | *phi081* | GAT-TAC | 6.05 | AAGGAACTGGTGAGAGGGTCCTT | AGCCCGATGCTCGCCATCTC |
| 98 | *phi102* | AT | 6.05 | TGAATCTAAACATAACTTATGTCTAGGTACATAGCAAA | CCTCGGATTCCGGATTGTAAGTCA |
| 99 | *bnlg1154* | (AG)27 | 6.05 | GGGTGATCACATGGGTTAGG | AAATCAATGCTCCAAATCGC |
| 100 | *bnlg1732* | (AG)15 | 6.05 | AACTTTTGGCATTGCACTGG | CGTAAGTGCACACGGCATTA |
| 101 | *bnlg1922* | (AG)17 | 6.05 | GTCTTGGGCAGTAATCAGGC | TCGATCAAAGACGTTCATGC |
| 102 | *umc2141* | (CT)8 | 6.05 | ATTAGCACCACCGTGTAGCAAGTT | GGCAGTGTGAGTGGTTGTGTG |
| 103 | *umc1296* | (GGT)7 | 6.06 | GCTGGAGATAGGCATCCAGACAC | CTCTCCCGGCTCTGACCTAGC |
| 104 | *umc2170* | (TAC)4 | 6.06 | CACTGCAAGCCTCTACAGACAATG | GAGAGTTCTCCAGGCGAGGTG |
| 105 | *umc2165* | (TTC)12 | 6.07 | AGAACACCAAATGGTGACGTTATGT | CTAGCTCGTCTTCCCTGTGGTCT |
| 106 | *bnlg1740* | (AG)21 | 6.07 | TTTTCTCCTTGAGTTCGTTCG | ACAGGCAGAGCTCTCACACA |
| 107 | *phi070* | AGCTG | 6.07 | GCTGAGCGATCAGTTCATCCAG | CCATGGCAGGGTCTCTCAAG |
| 108 | *phi299852* | AGC | 6.07 | GATGTGGGTGCTACGAGCC | AGATCTCGGAGCTCGGCTA |
| 109 | *phi089* | ATGC | 6.08 | GAATTGGGAACCAGACCACCCAA | ATTTCCATGGACCATGCCTCGTG |
| 110 | *umc2325* | (AGG)7 | 7.01 | CCTAGGAACTCTGATGGCTATGGA | CTACGATATCCACCTCTACCACCG |
| 111 | *umc1929* | NA | 7.02 | TCCAAGCATTATGCATCACAAACT | GCATGAATTCAACACAGAACCACT |
| 112 | *bnlg657* | NA | 7.02 | TCTGAGGATGCCCAATCATGCGC | CGTTTCCGTTCGTCACCAGCTCG |
| 113 | *bnlg1022* | (AG)12 | 7.02 | GTGTTGTCGATCCACTCCCT | GCAAAGATCTGTGAGGGGAC |
| 114 | *umc1036* | GA COMP. | 7.02 | CTGCTGCTCAAGGAGATGGAGA | GACACACATGCACGAGCAGACT |
| 115 | *umc1831* | (AG)8 | 7.02 | TTTCGACTGCTAGTGTACTTGGGG | CTCTACATCTTCAGCGTCTCCACA |
| 116 | *umc1978* | NA | 7.02 | CACTCCCTCCATTTCTCTCACCT | ATCGCTACCATTGGACGCTTTAC |
| 117 | *bnlg572* | NA | 7.03 | ACTGGACTGTCCTCGTGCCTA | CAAAAAAAGATTCGTTCGGAGTAA |
| 118 | *bnlg155* | NA | 7.03 | ACCGAGTAGCCGAGACACG | AGAGTCCTGGAGCCACATGAG |
| 119 | *phi114* | GCCT | 7.03 | CCGAGACCGTCAAGACCATCAA | AGCTCCAAACGATTCTGAACTCGC |
| 120 | *umc1456* | (AACC)5 | 7.03 | GCCACAGCTCACTAGCTCAAAAGT | CTCTGTGTGTTTGCTTGATTGCTT |
| 121 | *bnlg1161* | (AG)23 | 7.04 | GAACGGACGACGGTCGAT | ACCTCCACACGTCCCCAC |
| 122 | *umc1342* | AG(8) | 7.04 | TCTAATCCAATCGACATCGACAGA | TCGCCCTCTTTTCTTTTCTTTTCT |
| 123 | *bnlg1666* | (AG)34 | 7.04 | GCTGGTAGCTTTCAGATGGC | TGTCCCTCCTCCAGTTTCAC |
| 124 | *dupssr13* | (CA)12 | 7.04 | TCGTTCGGTCCATGAAAT | CAAATATCTCTCATCTTTGCTGAC |
| 125 | *umc2332* | (CTC)5 | 7.04 | GTCGGAGAAGGAGCTACTGAGCTA | CACAGGTACGTCTGGATGCTGT |
| 126 | *phi051* | AGG | 7.05 | CGACATCGTCAGATTATATTGCAGACCA | GGCGAAAGCGAACGACAACAATCTT |
| 127 | *umc2334* | (GGA)4 | 7.05 | ATGGCCTCCGTGCTGAAGAT | CATCTGATGGTGTTGTAGCAGCAG |
| 128 | *umc2190* | (CCT)4 | 7.06 | GATCCGTTGAGGTCGATCCTTT | GAGGAGTTCCTGCAGTTTCTTGAC |
| 129 | *umc1304* | (TCGA)4 | 8.02 | GCCAACTAGAACTACTGCTGCTCC | CATGCAGCTCTCCAAATTAAATCC |
| 130 | *umc1872* | (GCA)6 | 8.02 | CTTTTGTGATGTCTGCAATATGCC | TTAGTAGGTGCATTGGATGCTCAA |
| 131 | *umc2147* | (TCC)4 | 8.03 | CTACCCCTACGGCATCACCTACTT | AGACGGAAATGCACCGAGAAG |
| 132 | *phi115* | ATAC | 8.03 | GCTCCGTGTTTCGCCTGAA | ACCATCACCTGAATCCATCACA |
| 133 | *phi125* | AG | 8.03 | ACCGCCGGTGCGAGTTGAAG | CTTGGGATTGCCCTCATCCAC |
| 134 | *umc1627* | (GTAC)4 | 8.03 | TCATCATCATCATCATCTCCAACC | GACTCTATCCACCCATCTGTTCGT |
| 135 | *bnlg162* | NA | 8.05 | ACTAGCAGCAGTAAAACCTAATAAAGGGA | CAAGTAGCTAGCAGTCATTTGCAGTGT |
| 136 | *bnlg1812* | (AG)22 | 8.05 | CGAGAAGACTTGCGTGAACA | TTACGTGCGTCGTCAGAATC |
| 137 | *bnlg1599* | (AG)35 | 8.05 | CGAGAAGACTTGCGTGAACA | TTACGTGCGTCGTCAGAATC |
| 138 | *bnlg1607* | (AG)18 | 8.06 | CGTCCGTCCTTTTCTGAGAG | TTCGTCCAGATTTCATTCCC |
| 139 | *bnlg1823* | (AG)18 | 8.07 | TGTGACTCCATACCGCACAT | CTCATCATGTTGTACATGGCG |
| 140 | *phi080* | AGGAG | 8.08 | CACCCGATGCAACTTGCGTAGA | TCGTCACGTTCCACGACATCAC |
| 141 | *bnlg1272* | (AG)16 | 9.00 | ACCGAAGATGAGGTGTGACA | TCAGTGCAAGGGCAATTTAG |
| 142 | *phi028* | GAA | 9.01 | TCTCGCTGTCCTTCGATTAGTACGG | AATGCAGGCGATGGTTCTCCGGCCT |
| 143 | *umc2336* | (TGT)4 | 9.02 | CCTATGCTCTTGCTCTTCCTGGTA | ATCTCACCGCACGTAACTGAGACT |
| 144 | *bnlg1730* | (AG)26 | 9.03 | GGGTGCTCGTAGTAGGGGTT | AACACGTCAACAAGGGGAAG |
| 145 | *bnlg469* | NA | 9.03 | AGGGTGTACAGGTCCAAGTCCAA | AATGTGGGTCGTCAGCCATCAG |
| 146 | *phi022* | GTGC | 9.03 | TGCGCACCAGCGACTGACC | GCGGGCGACGCTTCCAAAC |
| 147 | *bnlg1209* | (AG)12 | 9.04 | GTCCCGGGCAGAATAATACC | TTCCTCCTTGAAGTGCTCGT |
| 148 | *umc1771* | (CGTC)4 | 9.04 | GTGAAATGTTGTTTCCAATGCAAG | CATCAGGAAGGAAGACGACTAGGA |
| 149 | *phi032* | AAAG | 9.04 | CTCCAGCAAGTGATGCGTGAC | GACACCCGGATCAATGATGGAAC |
| 150 | *umc1078* | (GT)13 | 9.05 | AGGCACTAGCAGGCGAGAGG | GCGTAGTAACATCCATCCAACCAA |
| 151 | *umc2341* | (TTC)6 | 9.05 | CTGAGCTCCTGATTTCTTGCTCTC | AAACATTTAATCCAACAGCCCAGA |
| 152 | *umc1231* | (GA)10 | 9.05 | CTGTAGGGCTGAGAAAAGAGAGGG | CGACAACTTAGGAGAACCATGGAG |
| 153 | *dupssr29* | (GA)24 | 9.07 | CAGCGAATACTGAATAACGC | TGTTGGATGAGCACTGAAC |
| 154 | *bnlg1129* | (AG)12 | 9.08 | GAGAGTATGCTACTCGCCGC | GACGAGTTTGGAGTGCCATT |
| 155 | *phi118* | AGG | 10.00 | GAAAGCGGAGAGAGGGCTTCAA | TTGGGATGTGATGTGAGAGCTTGCT |
| 156 | *umc1432* | (AG)16 | 10.02 | GGCCATGATACAGCAAGAAATGAT | TACTAGATGATGACTGACCCAGCG |
| 157 | *bnlg1451* | (AG)34 | 10.02 | TGATCGATGGCTCAATCAGT | ATCTGGAACACCGTCGTCTC |
| 158 | *phi059* | ACC | 10.02 | AAGCTAATTAAGGCCGGTCATCCC | TCCGTGTACTCGGCGGACTC |
| 159 | *umc2180* | (GGCC)4 | 10.03 | ATCAGCATCGATAGCGAAGAAAGA | ATTGCTACTAGGGTTGTTGTTGCC |
| 160 | *umc2017* | (CAA)4 | 10.03 | AGAGGTTACTACGGAGTGTGGCAG | GTCAGGGTACTGCTTCTCGAACTC |
| 161 | *phi050* | AAGC | 10.03 | TAACATGCCAGACACATACGGACAG | ATGGCTCTAGCGAAGCGTAGAG |
| 162 | *bnlg1518* | (AG)15 | 10.04 | AGCTGTACACGCAGTAGGCA | GGCTCTGTTAATTCGATCGC |
| 163 | *umc2156* | (TCG)5 | 10.04 | ACGACGGCAAGAAGAAAACTACTG | CGACCTGCTTTCAGTCTCTCTAGC |
| 164 | *umc1678* | (TCG)6 | 10.04 | GTAGAGATCGATTCGCTAACCTGC | AGTTGTTCCGTTCCGTCCTTATC |
| 165 | *umc2350* | (GGCCGT)4 | 10.04 | AGTAGCGACTCCTCTGCGTGAG | CGAATCGAGGATGGTTTGTTTTT |
| 166 | *bnlg1074* | (AG)14 | 10.05 | CATGCTAATAGCCTACCGGG | TTTCCCCCTGATTCGTTATG |
| 167 | *phi035* | AC | 10.06 | CGTGCAAGCAGTCCTCCCAG | CTCCCTGATGATGAGCTAGAAAGG |
| 168 | *bnlg236* | NA | 10.06 | CGCTTTGCAGTACCAGTACACAC | GACGACAACTGCAGAGTACCAGA |
| 169 | *bnlg1185* | (AG)24 | 10.07 | CGGTCCAGGCAGGTTAATTA | GACTCGAGGACACCGATTTC |
| 170 | *bnlg1360* | (AG)25 | 10.07 | TCTGCTCATCCACAACTTGC | AGAACGTGAAGCTGAGCGTT |
| 171 | *umc2083* | (CGG)7 | 1.05-1.06 | GATGCTCAAGGAGCAGCGAC | CAGGTGGTACGCCATGAACC |
| 172 | *umc1335* | (AG)24 | 1.06-1.07 | ATGGCATGCATGTGTTTGTTTTAC | ACAGACGTCGCTAATTCCTGAAAG |
| 173 | *umc2189* | (CAG)4 | 1.09-1.10 | CGTAAGTACAGTACACCAATGGGC | ACACCGACTACAAGCCTCTCAACT |
| 174 | *umc2363* | (ATGT)4 | 2.00-2.01 | TTGACTCGAAAGACTTGTGAGCTG | GTGTGTCAGAAGGAGGACTGATGA |
| 175 | *phi96100* | ACCT | 2.00-2.01 | AGGAGGACCCCAACTCCTG | TTGCACGAGCCATCGTAT |
| 176 | *umc1165* | (TA)6 | 2.00-2.01 | GTCGATTGATTTCCCGATGTTAAA | TATCTTCAGACCCAAACATCGTCC |
| 177 | *umc1552* | (GGA)7 | 2.01-2.02 | CTCGATAGCTCTGCTGCTTCCTC | CAACACCAGCCCTACCCAGA |
| 178 | *bnlg125* | NA | 2.02-2.03 | GGGACAAAAGAAGAAGCAGAG | GAAATGGGACAGAGACAGACAAT |
| 179 | *umc1746* | (CAC)4 | 3.00-3.01 | ACCTTGCCTGTCCTTCTTTCTCTT | ACACGAGCATCCTACATCCTCCTA |
| 180 | *umc2369* | (GCAC)4 | 3.02-3.03 | TTCGTCTGATGAAAGGTTCAGAGG | GATCCTCATCAAGACCAGCAGAGT |
| 181 | *bnlg1523* | (AG)17 | 3.02-3.03 | GAGCACAGCTAGGCAAAAGG | CTCGCACGCTCTCTCTTCTT |
| 182 | *umc2276* | (GGGC)4 | 3.08-3.09 | CTAGGTAGCCAGCTAGGTACGGGT | AGTGGAGCTTCTTCATCCTACCG |
| 183 | *umc1136* | (GCA)5 | 3.09-3.10 | CTGCATACAGACATCCAACCAAAG | CTCTCGTCTCATCACCTTTCCCT |
| 184 | *phi072* | AAAC | 4.00-4.01 | ACCGTGCATGATTAATTTCTCCAGCCTT | GACAGCGCGCAAATGGATTGAACT |
| 185 | *bnlg1126* | (AG)20 | 4.02-4.03 | GAGATCGAAGGTCATGGCAC | ATGGTTCCTGGTTCAGATGG |
| 186 | *umc2027* | (AAAG)4 | 4.05-4.06 | CAAATATCTTCGCAGCTCCAAATC | GTACCTGTCTTTGGGGCTTTTCTT |
| 187 | *umc2360* | (GCC)4 | 4.08-4.09 | TAGCAGCTAGCTTCAGTCACAGGC | CAGATCGGACTACTGGTGGCTAAG |
| 188 | *bnlg1879* | (AG)14 | 5.02-5.03 | TGCTCTCACAAGATGGTGGA | CCACAGGATAAAATCGGCTG |
| 189 | *umc1110* | (AGC)7 | 5.03-5.04 | TCTTGGAAGGCAAGACTCTACCTG | TTACACCAAGGTCCGAAACAAGAT |
| 190 | *phi113* | GTCT | 5.03-5.04 | GCTCCAGGTCGGAGATGTGA | CACAACACATCCAGTGACCAGAGT |
| 191 | *umc1482* | (AGC)5 | 5.04-5.05 | GAACAAAGAATCACAACACGATGC | CAGGTTCTGAGGAAAGCAAGGTT |
| 192 | *bnlg278* | NA | 5.05-5.06 | CATGCATCAACGTAACTCCCT | CATGTCACGCGTTCCACTTG |
| 193 | *bnlg118* | NA | 5.07-5.08 | CTTCCAGCCGCAACCCTC | CCAACAACGCGGACGTGA |
| 194 | *bnlg1617* | (AG)16 | 6.04-6.05 | CGTGCACGGTACAGAAAGAA | AGAAAGCCACGTACCCCTTT |
| 195 | *bnlg1136* | (AG)14 | 6.07-6.08 | TAACCGGATGAGCATCTTCC | CATCAGCTTCAACGAGTTCG |
| 196 | *umc1460* | (GCA)4 | 8.03-8.04 | GCTCAATCGTAGTAACAGCAGCAG | TCTGCACTAGAATGGCTTGGTACA |
| 197 | *phi014* | GGC | 8.04-8.05 | AGATGACCAGGGCCGTCAACGAC | CCAGCTTCACCAGCTTGCTCTTCGTG |
| 198 | *phi015* | AAAC | 8.08-8.09 | GCAACGTACCGTACCTTTCCGA | ACGCTGCATTCAATTACCGGGAAG |
| 199 | *umc2393* | (ACG)7 | 9.00-9.01 | CAACTCGATCCAGACCACACATAG | CTCTTGGTTGTTTGTTTCCTTGCT |
| 200 | *umc1743* | (GGC)4 | 9.03-9.04 | TGGACTTCGAAAATTCTCTTCAGC | GAGAGGAGGAGCTTCACGAGC |
| 201 | *bnlg1191* | (AG)39 | 9.06-9.07 | AATCATGCGTAGGCGTAGCT | GCCAGAGGAAAAAGAAGGCT |
| 202 | *bnlg1506* | (AG)19 | 9.07-9.08 | AAAGCCTCAGAGCTTCAACG | GCAGGCAACAACCAACAATA |
| 203 | *bnlg619* | NA | 9.07-9.08 | ACCCATCCCACTTTCCACCTCCTCCT | GCTTTCAGCGAATACTGAATAACGCGGA |
| 204 | *bnlg2137* | NA | Unassigned | - | - |
| 205 | *bnlg1578* | NA | Unassigned | - | - |
| 206 | *bnlg2387* | NA | Unassigned | - | - |
| 207 | *bnlg1789* | NA | Unassigned | - | - |
| 208 | *bnlg1852* | NA | Unassigned | - | - |

NA: Not Available
