# Supplementary material for: Development of Biofortified Maize Hybrids through Marker-Assisted Stacking of β-Carotene Hydroxylase, Lycopene-ε-Cyclase and Opaque2 Genes
Source: Front Plant Sci. 2018 Feb 20;9:178. doi: 10.3389/fpls.2018.00178 (PMC5826225; doi:10.3389/fpls.2018.00178)
Supplement: Table S4 — Morphological characterization of reconstituted hybrids along with their original versions. [file Table4.DOC]

Table S4. Morphological characterization of reconstituted hybrids along with their original versions

| **S. No.** | **Genotypes** | **MF**  **(days)** | | **FF**  **(days)** | | **PH**  **(cm)** | | **EH**  **(cm)** | | **EL**  **(cm)** | | **EW**  **(cm)** | | **NR**  **(no.)** | | **NKR**  **(no.)** | | **TW**  **(g)** | | **GY**  **(kg/ha)** | |
| --- | --- | --- | --- | --- | --- | --- | --- | --- | --- | --- | --- | --- | --- | --- | --- | --- | --- | --- | --- | --- | --- |
|  |  | **DL** | **DW** | **DL** | **DW** | **DL** | **DW** | **DL** | **DW** | **DL** | **DW** | **DL** | **DW** | **DL** | **DW** | **DL** | **DW** | **DL** | **DW** | **DL** | **DW** |
| 1. | HQPM1 | 61.0 | 57.0 | 62.0 | 59.0 | 180.0 | 181.5 | 80.0 | 83.8 | 15.0 | 15.2 | 3.7 | 3.6 | 14.3 | 14.3 | 31.0 | 25.0 | 24.2 | 26.3 | 6983 | 6135 |
| 2. | HQPM1-A | 58.5 | 55.5 | 60.5 | 59.0 | 180.0 | 180.6 | 77.5 | 81.3 | 15.5 | 15.7 | 3.8 | 3.8 | 14.0 | 14.0 | 33.3 | 27.3 | 24.1 | 29.7 | 7142 | 6398 |
| 3. | HQPM1-B | 59.0 | 56.0 | 60.0 | 59.0 | 190.0 | 181.9 | 75.0 | 86.3 | 16.1 | 14.9 | 3.9 | 3.3 | 14.7 | 14.3 | 33.2 | 23.2 | 26.9 | 27.9 | 7225 | 6461 |
| 4. | HQPM1-C | 60.5 | 56.5 | 62.0 | 59.0 | 172.5 | 176.9 | 75.0 | 78.1 | 15.6 | 15.0 | 3.8 | 4.0 | 14.0 | 14.8 | 32.7 | 27.3 | 24.7 | 28.1 | 7534 | 6301 |
| 5. | HQPM4 | 61.5 | 58.5 | 61.0 | 60.5 | 205.0 | 189.4 | 82.5 | 90.0 | 18.2 | 16.0 | 4.1 | 4.0 | 13.3 | 14.8 | 33.3 | 33.4 | 29.3 | 31.1 | 7360 | 7270 |
| 6. | HQPM4-A | 62.0 | 55.5 | 60.5 | 57.0 | 205.0 | 185.0 | 85.0 | 80.0 | 17.3 | 16.5 | 3.9 | 4.1 | 13.3 | 15.3 | 33.3 | 30.8 | 29.6 | 29.0 | 7618 | 7450 |
| 7. | HQPM4-B | 60.0 | 55.0 | 60.0 | 58.5 | 202.5 | 196.3 | 80.0 | 92.5 | 18.2 | 16.7 | 3.9 | 4.0 | 13.0 | 16.0 | 34.0 | 31.5 | 27.4 | 31.0 | 7234 | 7244 |
| 8. | HQPM4-C | 61.5 | 54.5 | 60.5 | 55.0 | 197.5 | 181.7 | 77.5 | 86.0 | 18.1 | 15.7 | 4.1 | 3.9 | 13.3 | 15.0 | 34.5 | 28.5 | 31.4 | 29.0 | 7975 | 6962 |
| 9. | HQPM5 | 59.5 | 57.0 | 58.5 | 60.5 | 185.0 | 176.9 | 75.0 | 77.5 | 16.4 | 14.4 | 4.0 | 3.4 | 13.7 | 15.7 | 31.8 | 24.5 | 27.1 | 28.6 | 8402 | 7185 |
| 10. | HQPM5-A | 57.5 | 55.5 | 56.5 | 58.0 | 202.5 | 191.9 | 77.5 | 90.1 | 15.7 | 15.8 | 3.9 | 4.0 | 14.0 | 15.7 | 33.3 | 28.5 | 28.0 | 29.8 | 8545 | 6668 |
| 11. | HQPM5-B | 60.5 | 56.0 | 59.0 | 59.0 | 190.0 | 182.5 | 82.5 | 86.9 | 16.5 | 14.8 | 4.1 | 3.9 | 13.3 | 14.7 | 32.8 | 29.0 | 27.2 | 27.0 | 8025 | 6651 |
| 12. | HQPM5-C | 60.5 | 56.5 | 59.0 | 60.0 | 190.0 | 192.1 | 80.0 | 88.1 | 16.0 | 15.0 | 4.0 | 4.3 | 14.0 | 16.7 | 30.7 | 27.8 | 27.2 | 26.1 | 7946 | 7510 |
| 13. | HQPM7 | 61.5 | 57.0 | 59.5 | 60.0 | 187.5 | 187.7 | 70.0 | 83.2 | 16.3 | 16.1 | 4.0 | 3.9 | 15.0 | 13.7 | 32.5 | 26.5 | 29.6 | 30.0 | 8479 | 6548 |
| 14. | HQPM7-A | 62.0 | 55.0 | 60.5 | 58.5 | 192.5 | 185.0 | 75.0 | 85.0 | 16.5 | 16.3 | 3.8 | 4.2 | 14.7 | 14.0 | 33.5 | 29.0 | 29.2 | 29.4 | 8181 | 7083 |
| 15. | HQPM7-B | 60.5 | 58.0 | 60.0 | 60.5 | 205.0 | 183.1 | 85.0 | 85.6 | 17.2 | 16.2 | 4.1 | 4.0 | 15.0 | 14.0 | 33.8 | 27.8 | 28.2 | 32.7 | 7842 | 6989 |
| 16. | HQPM7-C | 59.0 | 55.5 | 58.0 | 59.0 | 185.0 | 182.8 | 77.5 | 82.9 | 17.0 | 16.5 | 3.9 | 3.9 | 15.0 | 14.2 | 31.2 | 31.0 | 27.4 | 27.9 | 8283 | 6422 |
| **SE** | | **0.66** | **1.62** | **0.76** | **2.37** | **8.47** | **5.67** | **8.01** | **4.31** | **0.51** | **0.72** | **0.16** | **0.26** | **0.70** | **0.67** | **1.24** | **1.26** | **1.26** | **0.90** | **945** | **1106** |

**MF**: days to 50% male flowering; **FF**: days to 50% female flowering; **PH**: plant height; **EH**: ear height; **EL**: ear length; **EW**: ear width; **NR**: number of rows; **NKR**: number of kernels per row; **TW**: 100-seed weight; **GY**: grain yield; **DL**: Delhi; **DW**: Dharwad; **HQPM1-A**: HKI193-1-1-8-5-38 × HKI163-2-90-17-60; **HQPM1-B**: HKI193-1-1-8-5-116 × HKI163-2-90-10-7; **HQPM1-C**: HKI193-1-1-8-5-25 × HKI163-2-90-17-41; **HQPM4-A**: HKI193-2-10-8-34-68-34 × HKI161-24-62-53-61; **HQPM4-B**: HKI193-2-10-8-34-52-46 × HKI161-24-62-53-38; **HQPM4-C**: HKI193-2-10-8-34-52-46 × HKI161-24-62-53-61; **HQPM5-A**: HKI163-2-90-10-7 × HKI161-24-62-53-61; **HQPM5-B**: HKI163-2-90-10-7 × HKI161-24-62-53-67; **HQPM5-C**: HKI163-2-90-17-41 × HKI161-24-62-53-67; **HQPM7-A**: HKI193-1-1-8-5-116 × HKI161-24-62-53-61; **HQPM7-B**: HKI193-1-1-8-5-25 × HKI161-24-62-53-38; **HQPM7-C**: HKI193-1-1-8-5-38 × HKI161-24-62-53-61; **SE**: Standard Error
